# The pharmalogical reactivation of p53 function improves breast tumor cell lysis by granzyme B and NK cells through induction of autophagy

**DOI:** 10.1038/s41419-019-1950-1

**Published:** 2019-09-20

**Authors:** Marie Chollat-Namy, Thouraya Ben Safta-Saadoun, Djazia Haferssas, Guillaume Meurice, Salem Chouaib, Jerome Thiery

**Affiliations:** 1 INSERM U1186, Villejuif, France; 2 Gustave Roussy Cancer Campus, Villejuif, France; 3 University Paris Sud, Faculty of Medicine, Le Kremlin Bicêtre, France; 4 Bioinformatic Core Facility, INSERM US23/CNRS UMS3655, Gustave Roussy Cancer Campus, Villejuif, France; 5 Thumbay Research Institute of Precision Medicine, Gulf Medical University, Ajman, United Arab Emirates

**Keywords:** Breast cancer, Tumour immunology

## Abstract

Cytotoxic T lymphocytes (CTL) and natural killer cells (NK)-mediated elimination of tumor cells is mostly dependent on Granzyme B apoptotic pathway, which is regulated by the wild type (wt) p53 protein. Because *TP53* inactivating mutations, frequently found in human tumors, could interfere with Granzyme B-mediated cell death, the use of small molecules developed to reactivate wtp53 function in p53-mutated tumor cells could optimize their lysis by CTL or NK cells. Here, we show that the pharmalogical reactivation of a wt-like p53 function in p53-mutated breast cancer cells using the small molecule CP-31398 increases their sensitivity to NK-mediated lysis. This potentiation is dependent on p53-mediated induction of autophagy via the sestrin-AMPK-mTOR pathway and the ULK axis. This CP31398-induced autophagy sequestrates in autophagosomes several anti-apoptotic proteins, including Bcl-X_L_ and XIAP, facilitating Granzyme B-mediated mitochondrial outer membrane permeabilization, caspase-3 activation and Granzyme B- or NK cell-induced apoptosis. Together, our results define a new way to increase cytotoxic lymphocyte-mediated lysis of p53-mutated breast cancer cell, through a p53-dependent autophagy induction, with potential applications in combined immunotherapeutic approaches.

## Introduction

Cytotoxic T lymphocytes (CTL) and natural killer (NK) are important effector cells involved in the elimination of tumor cells, principally by releasing the contents of cytotoxic granules into the immune synapse^1^. The granule mediators of target cell lysis belong to the granzymes (Gzms) family, which induce cell death^2^ after their delivery into target cells by the pore-forming protein perforin (PFN)^3^. Human Granzyme B (GzmB), one of the principal mediators of this pathway, preferentially induces target apoptosis in a mitochondria-dependent manner. Indeed, human GzmB preferentially cleaves Bid, a BH3-only pro-apoptotic protein^4^. Truncated Bid (tBid) disrupts the mitochondrial outer membrane in a Bax/Bak-dependent manner, leading to the release of cytochrome c followed by caspases-9 and -3 activation and target cell apoptosis^4,5^. Importantly, tumor overexpression of pro-survival Bcl-2 family proteins, including Bcl-2, Bcl-X_L_, or Mcl-1^6–8^, can block this pathway by preventing mitochondrial outer membrane permeabilization (MOMP). Moreover, inhibitor of apoptosis (IAPs), such as survivin, XIAP, cIAP-1, or cIAP-2, can also block GzmB-induced apoptosis by preventing caspase-9 or -3 activation^9^.

The wild type (wt) p53 tumor suppressor normally protects cells from a variety of stress and the physiologic consequence of its activation essentially leads to growth arrest or apoptosis^10–12^, which are particularly important in order to maintain genome integrity^13^. Importantly, wtp53 activation can also positively or negatively regulate autophagy^14,15^, which is an intracellular self-digestive process that operates at basal level under normal circumstances but is increased under stressful conditions^16^. Even if numerous reports have implicated autophagy as a protective mechanism during cellular stress, autophagy can also participate to cell death induction or regulation^17^. Several p53-dependent mechanisms involved in autophagy induction have been described^18^ including Sestrin-1 and -2^19,20^ or unc-51-like kinase 1 (ULK1)^21^ transactivation. Sestrin-1 and -2 were found to facilitate the AMP-activated protein kinase (AMPK) phosphorylation, which in turn phosphorylates and activates the TSC1–TSC2 complex, thereby inhibiting the signaling of mammalian target of rapamycin (mTOR), a major autophagy inhibitor^19^. The serine/threonine protein kinase ULK-1, together with ULK-2, autophagy-related gene (ATG)-101, ATG13 and focal adhesion kinase family interacting protein of 200 kDa (FIP200) form the ULK complex which drives the phagophore formation, the initial autophagosomal precursor membrane structure^22^. On the opposite, several studies also demonstrated that p53 could stimulate anti-autophagic responses. For example, wtp53 proteins localized in the cytosol have an inhibitory effect on autophagy^15,23^, as gain-of-function mutant p53 proteins^24,25^.

Wtp53 has also recently emerged as an important regulator of the antitumor immune response at different levels^2,26^. In this regard, we demonstrated that wtp53 is an important positive modulator of GzmB-induced apoptosis^2,27–29^ and that siRNAs or drug-mediated inactivation of wtp53 significantly decrease target cells killing induced by cytotoxic effector cells or GzmB^27–29^. This suggests that p53 inactivating mutations frequently found in human tumors, especially in breast carcinoma, might be associated with a tumor cell resistance to killer lymphocyte-induced cell death^2^. Thus, the reactivation of wtp53 function in p53-mutated tumor cells could be a novel strategy to optimize CTL/NK-mediated killing. For this purpose, several novel molecules were identified during the past decade based on their properties to restore wtp53 conformation and function of mutant p53 proteins (e.g. CP-31398, APR-246…)^30–32^. As a proof of principles, the aim of this study was thus to investigate in vitro the effect of one of these p53-reactivating molecules on GzmB- and NK cell-mediated killing of p53-mutated breast tumor cells. We found that the reactivation of wtp53 activity using CP-31398 potentiates NK- and GzmB-mediated lysis of p53-mutated breast carcinoma cells. Mechanistically, we observed that CP-31398 induces autophagy in a p53-dependent manner through the induction of Sestrin-1 and -2 expression, leading to AMPK activation and mTOR inhibition, as well through the induction of ULK-1. We also found that CP-31398-induced autophagy is blocked at the autophagosomes/lysosomes fusion step, but is sufficient to facilitate GzmB- and NK cell-induced MOMP and caspase-3 cleavage in target cells, mostly though the sequestration of the anti-apoptotic proteins Bcl-X_L_ and XIAP in autophagosomes.

## Materials and methods

### Cell lines

MDA-MB231 (NCI-DTP Cat# MDA-MB-231, RRID:CVCL_0062), T47D (NCI-DTP Cat# T-47D, RRID:CVCL_0553), SKBR3 (ATCC Cat# HTB-30, RRID:CVCL_0033), and MCF7 (NCI-DTP Cat# MCF7, RRID:CVCL_0031) cell lines were grown in RPMI-1640/Glutamax™ (Gibco) supplemented with 10% FBS and 100 U/ml penicillin/streptomycin (Gibco). The NK92 (ATCC Cat# PTA-6670, RRID:CVCL_2142) cell line and NK cells isolated from healthy donors (NKd) by positive selection (CD56 Positive Selection Kit; Stem Cell) were cultured in RPMI 1640/GlutaMax™ (Gibco) supplemented with 10% FBS and 300 U/mL rhIL2. All cell lines were originally obtained from ATCC, tested for *Mycoplasma* infection by PCR (Venor GeM OneStep; Minerva Biolabs) every 3–6 months and used at low passage (<50).

### CP-31398 treatment

Cells were treated with 7.5 μg/mL CP-31389 (Sigma-Aldrich) during 24 or 48 h. In some cases, tumor cells were co-treated with 20 µM PFT-α (Sigma-Aldrich) during 48 h or with 50 µM Chloroquine (Sigma-Aldrich) during 16 h.

### Antibodies

The following antibodies were used for either western blot, immunoprecipitation, or flow cytometry analysis:

*From Santa Cruz Bio:* anti-p53 mouse mAb (clone DO-1), anti-Bcl-2 mouse mAb (clone 100), anti-Bcl-X_L_ mouse mAb (clone H-5), anti-Sestrin-2 mouse mAb (clone 41-K), anti-survivin mouse mAb (clone D-8), anti-Beclin1 mouse mAb (clone E-8), anti-cIAP-1 rabbit pAb (clone H-83), anti-cIAP-2 rabbit pAb (clone H-85), anti-Mcl-1 rabbit pAb (clone S-19), anti-Bax mouse mAb (clone N20). *From Cell Signaling:* anti-caspase 3 (p35) mouse mAb (clone 3G2), anti-cleaved caspase 3 (p19/p17) rabbit mAb (clone 5A1E), anti-PARP rabbit pAb (cat #9542), anti-Atg101 rabbit mAb (clone E1Z4w), anti-Atg13 rabbit mAb (clone D4P1K), anti-FIP200 rabbit mAb (clone D10D11), anti-ULK1 rabbit mAb (clone D8H5), anti-AMPKα rabbit mAb (clone D5A2), anti-Phospho-AMPKα (Thr172) rabbit mAb (clone D4D6D), anti-mTOR rabbit pAb (cat #2972), anti-Phospho-mTOR (Ser2448) rabbit pAb (cat #2971), anti-LC3B rabbit pAb (cat #2775), anti-Atg5 rabbit mAb (clone D5F5U), anti-Bid rabbit mAb (cat #2002). *From BD Bioscience:* anti-XIAP mouse mAb (clone 28/hILP). *From Merck:* anti-p21 mouse mAb (clone OP64), anti-mdm2 mouse mAb (clone 2A10). *From StressGen:* anti-mtHsp70/Grp75 mouse mAb (cat #SPA-810B). From Abcam: anti-Sestrin-1 mouse pAb (cat #ab67156). *From Sigma-Aldrich:* HRP-conjugated anti-actin mouse mAb (clone AC-74), anti-p62 rabbit pAb (cat #P0067). *From ProteinTech:* anti-NBR1 rabbit pAb (cat #16004-1-AP). *From Beckman Coulter:* FITC-conjugated anti-Fas/CD95 mouse mAb (clone UB2). *From Bio Legend:* PE-conjugated anti-DR4/CD261 mouse mAb (clone DJR1), PE-conjugated anti-DR5/CD262 mouse mAb (clone DJR2-4 (7-8)). *From R&D System:* PE-conjugated anti-ULBP-1 mouse mAb (clone 170818); APC-conjugated anti-ULBP-2/5/6 mouse mAb (clone 165903); PE-conjugated anti-ULBP-3 mouse mAb (clone 166510); PE-conjugated anti-ULBP-4 mouse mAb (clone 709116). *Generated in the laboratory:* anti-ICAM-1 (clone 25D7) mouse mAb, anti-MHC-class I mouse mAb (clone MA2.1).

### RNA isolation and cDNA synthesis

Total RNAs were extracted from cell samples using Trizol solution (Invitrogen). The quality of RNAs was assessed using a Bio-Analyzer instrument (Agilent) and then quantified using a BioSpecNano (Shimadzu Biotech). cDNA synthesis was performed with 1 μg total RNA and a Maxima First Strand cDNA Synthesis Kit (Thermo Scientific).

### RT-qPCR analysis

Gene expression was quantified by SYBR Green qPCR method using SYBR Select Master Mix on a StepOnePlus Real Time PCR system (Thermo Scientific). Relative expression was calculated using the comparative Ct method (2-ΔCt). Transcript level of 18S was used as endogenous control. Primers were purchased from Sigma-Aldrich and their sequences (FW (5′ → 3′)--RV (5′ → 3′)) are listed below:

CDKN1A (p21): ctgccgaagtcagttccttgt--catgggttvtgacggacatc

ULK1: gtcacacgccacataacag--tcttctaagtccaagcaca

ULK2: tttaaatacagaacgaccaatgga--ggaggtgccagaacacca

ATG5: caacttgtttcacgctatatcagg--cactttgtcagttaccaacgtca

GABARAPL2: ccgtcgttgttgttgtgct--ctccacgcatctgtgttcc

ZFYVE1: atccccgatgaccacatg--tcatgcttttcttacatccaacc

ATG12: cataaaaacacttagagcaaactacca--cagataaaaaccagaataactggaca

ATG13: agctgccttgatctgactgg--ataccccggggctcttcta

SESN-1: tggactctgcagcagagatt--ctgatggacgatgaggtgtt

SESN-2: tgcctcctctctgaccagtt--cctcttctctcctgcacacc

ATG101: gaagtgtggacggtcaaggt--cacgttatccacctccgact

FIP200: cagatgctgaaagtggcaaa--ggcaatagtttgacggcatt

### Microarray assay, data processing, and analysis

Quadruplicate samples from untreated (24–48 h) compared to CP-31398-treated (24–48 h) MDA-MB231 cells were analyzed. Gene expression analysis was performed with Agilent® SurePrint G3 Human GE 8 × 60K Microarray (Agilent Technologies, AMADID39494). Samples were labeled with Cy3/Cy5 using a two-color Agilent labeling kit (Low Input Quick Amp Labeling Kit; cat #5190-2306) adapted for small amount of total RNA (100 ng total RNA per reaction). Hybridization was then performed on microarray using linearly amplified labeled cRNA, following the manufacturer protocol and Agilent SureHyb Chamber. After washing in acetonitrile, slides were scanned using an Agilent G2565CA microarray scanner with defaults parameters. Microarray images were analyzed using Feature Extraction software (FES; version 10.7.3.1) from Agilent technologies. Defaults settings were used. For the data processing and analysis, raw data files from FES were imported into R with LIMMA (an R package from the Bioconductor project)^33^, and processed as follow: gMedianSignal data were imported, controls probes were systematically removed, and flagged probes (gIsSaturated, gIsFeatpopnOL, gIsFeatNonUnifOL) were set to NA. Inter-array normalization was performed by quantile normalization. To get a single value for each transcript, we took the mean of each replicated probes summarized data. Missing values were inferred using KNN algorithm from the package “impute” from R bioconductor. Normalized data were then analyzed. To assess differentially expressed genes between two groups, we start by fitting a linear model to the data. Then we used an empirical Bayes method to moderate the standard errors of the estimated log-fold changes. The top-ranked genes were selected based on an absolute fold-change >2 and an adjusted *p*-value (FDR) <0.05. Data have been deposited in the ArrayExpress database at EMBL-EBI (www.ebi.ac.uk/arrayexpress) under accession number E-MTAB-8143.

### Western blot

Total protein extracts were prepared from cells treated or not with CP-31398 during the indicated time, γ-irradiated (5 Gy) or co-incubated with NKd cells at the indicated effector:target ratio during 30 min, lysed by RIPA buffer containing a cocktail of protease and phosphatase inhibitors (Thermo Scientific). Proteins were denatured 10 min at 95 °C in Laemmli buffer before SDS-PAGE separation on 4–20% precast gels (BioRad) and transfer on nitrocellulose membrane (Thermo Scientific). Blots were probed with the indicated primary Ab and HRP-conjugated secondary Ab. Western blot quantification was performed using the Image-J densitometry software.

### NBR1 and p62 immunoprecipitation

Following treatment with CP-31398, NBR1, or p62 immunoprecipitation was performed overnight at 4 °C using a classic IP kit (Pierce) and anti-p62 (cat #P0067; Sigma) or anti-NBR1 (cat #16004-1-AP; ProteinTech) rabbit pAbs, according to the manufacturer’s instructions. Immune complexes were eluted and washed before boiling in Laemmli buffer. Immunoprecipitates and cell lysates (input) were run in SDS-PAGE gel (4–20% precast gel, Biorad) and immunoblotted with HRP-conjugated anti-Bcl-2, anti-BclXL, anti-XIAP, anti-LC3, anti-NBR1, or anti-p62 Abs.

### Flow cytometry analysis of autophagy

To evaluate autophagy induction, MDA-MB231 ± CP-31398 (48 h) were incubated for 30 min at 37 °C in dilution buffer containing Cyto-ID-green (Cyto-ID Autophagy Detection Kit; Enzo Life Science) according to the manufacturer’s instructions. Cells were then washed with dilution buffer and immediately analyzed with a BD Accuri™ C6 flow cytometer. Data analysis was performed with the FlowJo software.

### Flow cytometry analysis of cell surface markers

To analyze FAS, DR4, DR5, ULBP-1, ULBP-2/5/6, ULBP-3, ULBP-4, ICAM-1, or MHC-Class I expression at the cell surface, MDA-MB231 ± CP-31398 cells were incubated 30 min at 4 °C with appropriate isotype control or one of the specific antibody indicated above. In some case, a secondary antibody conjugated to Alexa Fluor®488 (Life Technologies) was used. Cells were then fixed with PBS 1X containing 2% formaldehyde (PolySciences Inc.) before analysis with a BD Accuri™ C6 flow cytometer. Data analysis was performed with the FlowJo software.

### Formation of effector /target cell conjugates

MDA-MB231 cells ± CP-31398 and NKd cells were respectively labeled during 10 min at 37 °C with the lypophilic dyes DiO or DiD (Life Technologies) and washed three times. Target cells were then incubated with NKd at the ratio effector:target of 3:1 in Ca^2+^ free medium (HBSS containing 10 mM of Hepes pH 7,5 and 0.4% BSA) during 20 min at 37 °C to allow conjugates formation before flow cytometry analysis (BD Accuri™ C6). The percentage of DiO^+^ MDA-MB231 cells conjugated to DiD^+^ NKd, corresponding to immune complex, was quantified with the FlowJo software.

### Fluorescence microscopy analysis

MDA-MB231 cells stably transfected with a vector encoding Tomato-LC3 using FuGENE (Promega) were grown on collagen-coated glass coverslips (Sigma). After 48 h ± CP-31398 treatment, cells were fixed and stained with the indicated primary and secondary Ab conjugated to Alexa Fluor®488 or 647 (Life Technologies). Coverslips were mounted with Vectashield/DAPI (Vector Laboratories). Image acquisition was performed with a IX83 epifluorescence microscope and data analyzed with CellSens Dimension software (Olympus). The colocalization between Tomato-LC3^+^ autophagosomes and the protein of interest was measured and is displayed as a pearson’s correlation coefficient R(r) from at least 100 cells from three independent experiments. The number of autophagosome per cell was also evaluated in at least 250 cells from three independent experiments.

### Transmission electron microscopy

Cells were incubated with or without CP-31398 for 48 h or CQ for 16 h. Cell pellet were then fixed for 1 hr at 4 °C in 2% glutaraldehyde in 0.1 M Sörensen phosphate buffer, pH 7.3. Cells pellets were post-fixed for 1 h with aqueous 2% osmium tetroxide. Cells were then treated with 2% uranyl acetate in 30% methanol, dehydrated and embedded in Epoxy Embedding Medium (Sigma). Polymerization was carried out for 48 h at 65 °C. Ultrathin sections were collected and contrasted by uranyl acetate and lead citrate. For analysis, cells were observed with a FEI Tecnai 12 electron microscope using a CCD Megaview III camera and a SIS system (Olympus Soft Imaging Solutions GmbH).

### Monitoring of the autophagosome to autolysosome maturation by tandem sensor

The fusion between autophagosomes and autolysosomes was measured with a Premo Autophagy Tandem Sensor RFP-eGFP-LC3B kit (Thermo Scientific) according to the manufacturer’s instructions. Briefly, MDA-MB231 cells were seeded in a 6-well culture plate and infected with LC3B BacMam reagent (30 viral particles per cell) during 24 h. Cells were then treated with chloroquine (16 h) or CP-31398 (48 h). To induce starvation, cells were cultured 24 h in serum-free medium. Cells were then analyzed with a IX83 microscope and CellSens Dimension software (Olympus). Autophagosomes (in yellow) and autolysosomes (in red) were counted in at least 100 infected cells from three independent experiments.

### siRNAs transfection

Subconfluent MDA-M231 cells were transfected twice, at a 24 h interval, with the following siRNAs using Lipofectamine RNAiMAX (Invitrogen): controls siRNA (Ambion Silencer Select ID:4390843), Beclin-1 siRNA (Ambion Silencer Select ID:S16537), ATG5 siRNA (Qiagen APG5L_6 FlexiTube siRNA, cat #SI02655310), AMPKα siRNA (Sigma Mission® esiRNA, cat# EHU074041) or p53 siRNA (Ambion Silencer Select 4390824 ID:s607). Twenty-four hour after the second transfection, cells were treated or not with CP-31398 and incubated during 48 h before analysis.

### Measurement of CP-31398-induced apoptosis

To assess CP-31398-induced apoptosis, MDA-MB231 cells ± CP-31398 (48 h) were resuspended in Annexin Buffer (150 mM NaCl, 5 mM KCl, 1 mM MgCl_2_, 1.8 mM CaCl_2_, 10 mM Hepes pH 7.4) containing APC-conjugated annexin V (Thermo Scientific). After 10 min incubation at room temperature, Annexin Buffer containing 10 μg/ml propidium iodide (Sigma-Aldrich) was added. Apoptosis was immediately measured by flow cytometry (BD Accuri™ C6).

### ROS monitoring by flow cytometry

To measure intracellular ROS production, MDA-MB231 ± CP-31398 (48 h) were incubated for 7 min in PBS containing 10 µM oxidation-sensitive fluoroprobe Dihydroethidium (Thermo Scientific) according to the manufacturer’s instructions. A 30 min treatment with 1% H_2_O_2_ was used as positive control. Cells were then immediately analyzed by flow cytometry (BD Accuri™ C6).

### Measurement of mitochondrial outer membrane permeabilisation (MOMP)

MDA-MB231 cells ± CP-31398 and pre-labeled with Vybrant DiD (Life Technologies) were incubated 30 min with NKd (effector/target ratio 5:1) or 30 min with PFN ± GzmB and then 15 min at 37 °C with 3,3’-dihexyloxacarbocyanine iodide (DioC6(3)) (Life Technologies). Labeled cells were then washed, resuspended in PBS and analyzed immediately by flow cytometry (BD Accuri™ C6), focusing on DiD^+^ cells.

### Treatment with PFN and GzmB or GzmA

Native rat PFN was purified from RNK16 cells and native hGzmB was purified from YT-Indy cells as described^34^. GzmA was purified as described^35^. MDA-MB231 cells ± CP-31398 were resuspended in HBSS (Gibco) containing 10 mM Hepes pH 7.5, 4 mM CaCl_2_, 0.4% BSA before adding sublytic dose of PFN ± 100 nM hGzmB or 500 nM GzmA, diluted in HBSS containing 10 mM Hepes pH7.5. To assess PFN/GzmB-induced apoptosis, cells incubated for 2 h at 37 °C with buffer or sublytic PFN ± 100 nM hGzmB were analyzed for caspase activation by flow cytometry using M30-FITC mAb staining according to the manufacturer protocol (M30 CytoDEATH, Roche) to detect an effector caspase-cleavage product of cytokeratin-18^36^. To assess PFN/GzmA-induced cell death, cell were analyzed by flow cytometry after Annexin-V/PI staining, as described above.

### Chromium release assay

NK cell cytotoxic activity was measured by a 4 h ^51^Cr release assay performed in triplicate, as previously described^27^. Data are expressed as the percentage of specific ^51^Cr release from target cells, calculated as (experimental release-spontaneous release)/(maximum release-spontaneous release) × 100. Inhibition of the PFN/Gzm-mediated cytotoxic pathway was performed by pre-incubating NK cells for 2 h with 100 nM concanamycin A (CMA)(Sigma-Aldrich). Functional effects of ZB4 anti-Fas neutralizing mAb was also tested by pre-incubating target cells for 2 h before the assay.

### Statistical analysis

Data are expressed as mean from at least three independent experiments ± standard deviation (s.d.). For comparisons between two groups, Student's *t* test was used. For analysis with multiple comparisons, one-way analysis of variance (ANOVA) was performed. *P* values < 0.05 were considered as statistically significant.

## Results

### CP-31398 reactivates a wild type-like p53 transcriptional activity in p53-mutated breast tumor cells and increases their lysis by NK cells

**Fig. 1:**
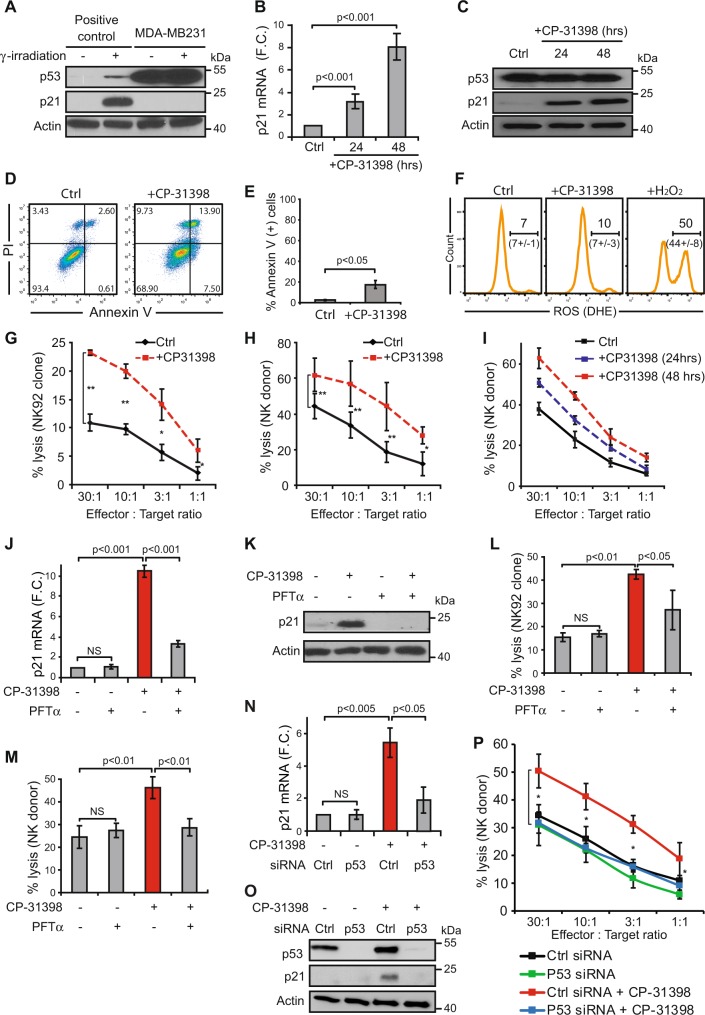
The reactivation of p53 transcriptional activity by CP-31398 increases p53-mutated breast cancer cell susceptibility to NK-mediated lysis. **a** MDA-MB231 cells express high level of mutated p53 (p53^R280K^) lacking transcriptional activity as shown by the absence of p53-dependent transactivation of its target gene p21 even after γ-irradiation. **b**, **c** 24-48 h treatment with CP-31398 reactivates p53 transcriptional activity in MDA-MB231 cells as shown by induction of p21 expression at both mRNA (**b**) and protein (**c**) levels. **d**–**f** CP-31398-induced apoptosis and ROS production were evaluated after 48 h treatment using AnnexinV/PI (**d**, **e**) or DHE (**f**) staining. **g**, **h** CP-31398 treatment (48 h) increases MDA-MB231 cell susceptibility to NK-mediated lysis. ^51^Cr release assay using NK92 (**d**) or NK cells isolated from a healthy donor (**e**) co-cultured with target cells at different E:T ratios are shown. **i** CP-31398 treatment increases MDA-MB231 cell susceptibility to NK-mediated lysis in a time dependent manner. A representative ^51^Cr release assay (from two independent experiments) using NK cells isolated from a healthy donor is shown. **j**, **k** PFT-α (an inhibitor of p53 transcriptional activity) inhibits CP-31398-dependent induction of p21 expression in MDA-MB231 cells at both mRNA (**j**) and protein (**k**) levels. **l**, **m** PFT-α inhibits the increase of MDA-MB231 lysis by NK cells observed when CP-31398 is used alone. ^51^Cr release assay using NK92 (**l**) or NK cells extracted from a healthy donor (**m**) co-cultured with target cells at the E:T ratio of 50:1 or 30:1, respectively, are shown. **n**–**p** The knockdown of mutated p53 expression using siRNA inhibits CP-31398-dependent induction of p21 expression in MDA-MB231 cells at both mRNA (**n**) and protein (**o**) levels and abrogate the CP-31398-dependent increase of MDA-MB231 lysis by NK cells (**p**). Data are representative of at least three independent experiments (**a**, **c**, **d**, **k**, **o**) or are the mean ± s.d. of three (**e**–**h**, **l**, **m**, **p**) or five (**b**, **j**, **n**) independent experiments. The p values (*P < 0.05; **P < 0.01) were determined by unpaired two-tailed Student's *t* test (**b**, **e**, **g**, **h**, **j**, **l**–**n**) or one-way ANOVA (**p**)

To evaluate the effect of wtp53 function reactivation on cytotoxic lymphocyte-mediated lysis of cells harboring a mutated p53, we used CP-31398^37^ and the breast carcinoma cell line MDA-MB231. As previously described, these cells express a mutated p53^R280K^ lacking transcriptional activity as shown by the absence of p21 transactivation even after γ-irradiation (Fig. 1a). As expected, MDA-MB231 cells treatment with CP-31398 during 24-48 h induces p21 mRNA and protein expression (Fig. 1b, c), suggesting that CP-31398 reactivates p53^R280K^ transcriptional capacities, with only a slight increase of cell death (Fig. 1d, e) and no detectable reactive oxygen species (ROS) production (Fig. 1f). Noteworthy, the lysis of MDA-MB231 cells by NK92 or NK cells isolated from a healthy donor (NKd) was strongly increased after CP-31398 treatment (Fig. 1g, h), in a time dependent manner (Fig. 1i). Similar results were obtained using the breast tumor cells T47D (p53^L194F^) and SKBR3 (p53^R175H^) (Supplementary figure S1A–G), while CP-31398 has no effect on MCF7 (p53^WT^) lysis by NK cells (Supplementary figure S1H–J). We next used CP-31398 in combination with pifithrin-α (PFT-α), which mainly blocks wtp53 transcriptional activity^38^. As shown in Fig. 1j, k, PFT-α inhibits CP-31398-dependent expression of p21 at both mRNA and protein levels, and almost completely inhibits the CP-31398-mediated increase of MDA-MB231 lysis by NK cells (Fig. 1l, m). Similar results were also obtained after the inhibition of mutant p53 expression using siRNAs (Fig. 1n–p). Taken together, these data demonstrate that CP-31398 can increase the lysis of p53-mutated tumor cells by killer cells, probably by reactivating p53 transcriptional activity.

### CP-31398-dependent reactivation of wild type-like p53 transcriptional activity induces the expression of autophagy-related genes involved in autophagy initiation

**Fig. 2:**
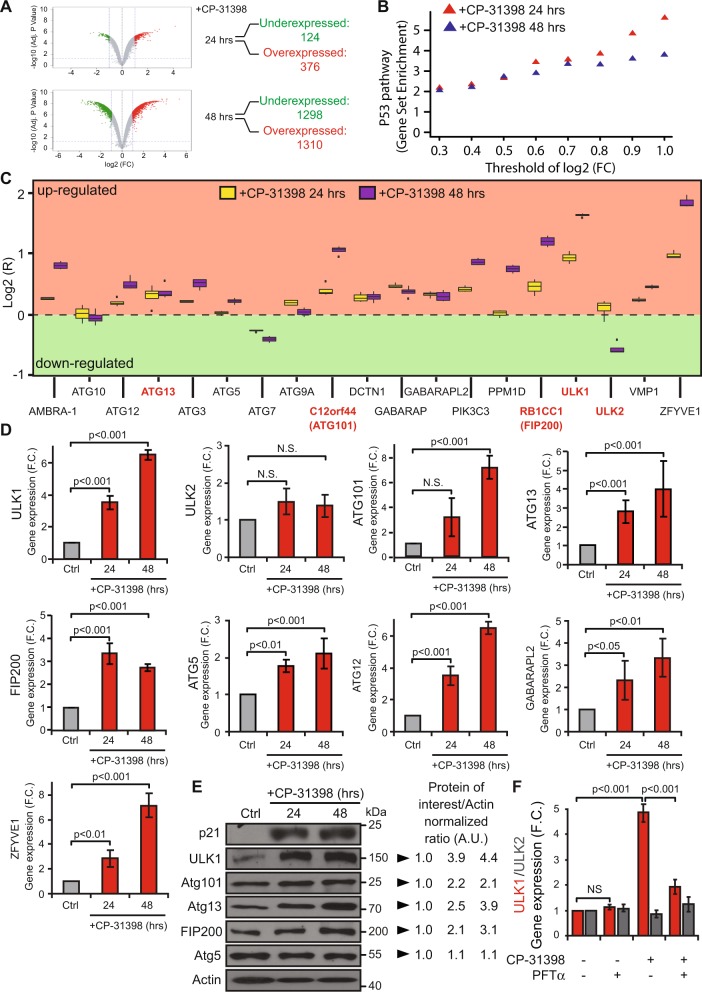
The reactivation of p53 transcriptional activity by CP-31398 leads to the overexpression of genes belonging to the ULK complex and involved in autophagy initiation. **a** Volcano-plot obtained from microarray analysis of MDA-MB231 cells treated with CP-31398. The number of overexpressed and downregulated genes (F.C. > 2; *p* < 0.05) is indicated. **b** Genes belonging to the p53 pathway/network (hallmark_P53_pathway; GSEA) are enriched in overexpressed genes following CP-31398 treatment (24 and 48 h). **c** Targeted analysis of microarray results using a pre-established list of genes involved in autophagosomes formation. Most of these genes are upregulated following 24–48 h CP-31398 treatment. Genes indicated in red encode proteins belonging to the ULK complex. **d** RT-qPCR validation of *ULK1, ULK2*, *ATG101*, *ATG13*, *FIP200*, *ATG5, ATG12, GABARAPL2*, and *ZFYVE1* expression following CP-31398 treatment (24 and 48 h) compared to untreated cells (F.C. fold-change). **e** CP-31398 treatment (24–48 h) increases the expression of several proteins belonging to the ULK complex (ULK1, ATG101, ATG13, FIP200). ATG5 was used as a control. The normalized ratio protein of interest/Actin was calculated by densitometry analysis and display. Data are representative of three independent experiments. **f** PFT-α (an inhibitor of p53 transcriptional activity) inhibits CP-31398-dependent induction of *ULK1* expression, while *ULK2* expression is not affected. Data (**d**, **f**) are the mean ± s.d. of five independent RT-qPCR experiments performed in duplicate. The *p* values (**d**, **f**) were determined by unpaired two-tailed Student's *t* test. (*NS* non-significant)

To understand how the reactivation of p53 transcriptional activity in p53-mutated cells increases their lysis by NK cells, we first examined whether CP-31398 treatment modified the expression of known p53 targets related to NK cell activity. No significant differences were observed for ICAM-1 expression (which interacts with LFA-1 and contributes to NK cells adhesion to target cells) or for the formation of immune conjugates between NKd and MDA-MB231 cells (Supplementary figure S2A–C). Similarly, the expression of MHC-class I, ULBP1-6 (the ligands of the activating receptor NKG2D), or death receptors (FAS, DR4/DR5) were unmodified by CP-31398 (Supplementary figure S2D–G). Thus, to analyze more deeply the effect of CP-31398 treatment on MDA-MB231 cells that could explain its effect on their susceptibility to NK cell-mediated lysis, and since CP-31398 effect relies on the reactivation of p53 transcriptional activity; we next performed a transcriptomic analysis of MDA-MB231 cells treated during 24–48 h with CP-31398. The data obtained showed that several genes are overexpressed and downregulated after 24–48 h CP-31398 treatment (Fig. 2a), with an enrichment of overexpressed genes belonging to the p53 pathway (hallmark_P53_pathway; GSEA; M5939), as expected (Fig. 2b). Furthermore, by a targeted analysis using a pre-established list of genes involved in autophagosome formation (establish by Mizushima group^39^), we observed a global overexpression after CP-31398 treatment (Fig. 2c). RT-qPCR validation confirmed that expression of *ULK1*, *ATG101*, *ATG13*, and *FIP200* (involved in the ULK complex formation and in early steps of autophagosome biogenesis), *ATG5*, *ATG12*, and *GABARAPL2* (involved in autophagosomal membrane elongation) and *ZFYVE1* (involved in autophagy initiation) were increased after CP-31398 treatment (Fig. 2d). Interestingly, ULK1 protein level, previously described as a direct p53 target^21^, was also highly increased by CP-31398 treatment, as ATG101, ATG13, and FIP200 but to a lower extend (Fig. 2e). Moreover, the combination of PFT-α with CP-31398 decrease CP-31398-induced *ULK1* overexpression, while *ULK2* expression is not affected (Fig. 2f). Taken together, these results suggest that the reactivation of p53 transcriptional activity by CP-31398 could initiate autophagy by upregulating several proteins involved in early steps of autophagosome biogenesis, especially ULK1.

### CP-31398-dependent reactivation of wild type-like p53 transcriptional activity activates the Sestrin-AMPK pathway resulting in mTOR inhibition

**Fig. 3:**
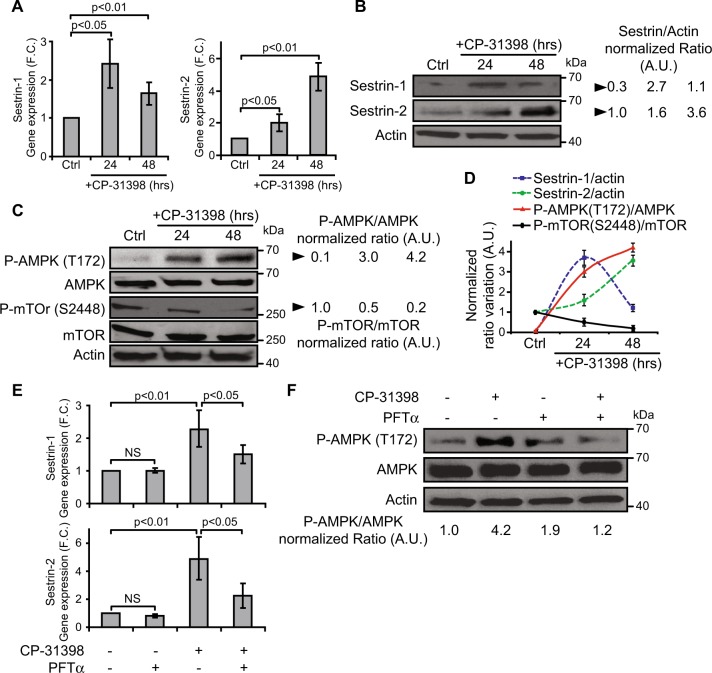
The reactivation of p53 transcriptional activity by CP-31398 leads to the activation of the Sestrin/AMPK pathway and to the inhibition of mTOR. **a**, **b** CP-31398 treatment of MDA-MB231 cells increases Sestrin-1 and -2 expression at both mRNA (**a**) and protein (**b**) levels. **c** CP-31398 treatment activates AMPK phosphorylation (T172) and decrease phosphorylated m-TOR (S2448) level. **d** The normalized ratio of Sestrin-1 or -2/Actin, P-AMPK/AMPK, P-mTOR/m-TOR were calculated by densitometry analysis and display (**e–f**) PFT-α (an inhibitor of p53 transcriptional activity) inhibits CP-31398-induced *Sestrin-1* and *−2* transcriptional activation (**e**) and AMPK phosphorylation (T172) (**f**). Data are representative of three independent experiments (**b**, **c**, **f**) or are the mean ± s.d. of three independent RT-qPCR experiments (**a**, **d**, **e**). The *p* values (**a**, **e**) were determined by unpaired two-tailed Student's *t* test

The reactivation p53 transcriptional activity could also induce autophagy by increasing the expression of other key genes, not included in the pre-established list of genes used to analyze the microarray data. This includes *Sestrin-1* and *Sestrin-2*, two known p53 targets^19^, also found upregulated in our transcriptomic results (0.5 ≥ FC ≥ 2 after 24–48 h CP-31398 treatment). As expected, MDA-MB231 cells treatment with CP-31398 during 24-48 h induces Sestrin-1 and Sestrin-2 expression at both mRNA and protein levels (Fig. 3a, b). Consequently, CP-31398 induces the phosphorylation of AMPK (Thr172), involved in AMPK activation, and the dephosphorylation of mTOR (S2448) (Fig. 3c, d), which inhibits mTOR activity and consequently could induce autophagy^22^. Finally, MDA-MB231 cells treatment with PFT-α inhibits both *Sestrin-1* and *Sestrin-2* overexpression and AMPK phosphorylation (Thr172) induced by CP-31398 (Fig. 3e, f). Together, these data show that CP-31398 modulates the AMPK/mTOR axis at a post-translational level through the transcriptional induction of Sestrin-1 and Sestrin-2, which could also initiate autophagy.

### CP-31398-dependent reactivation of p53 induces the formation of autophagosomes

**Fig. 4:**
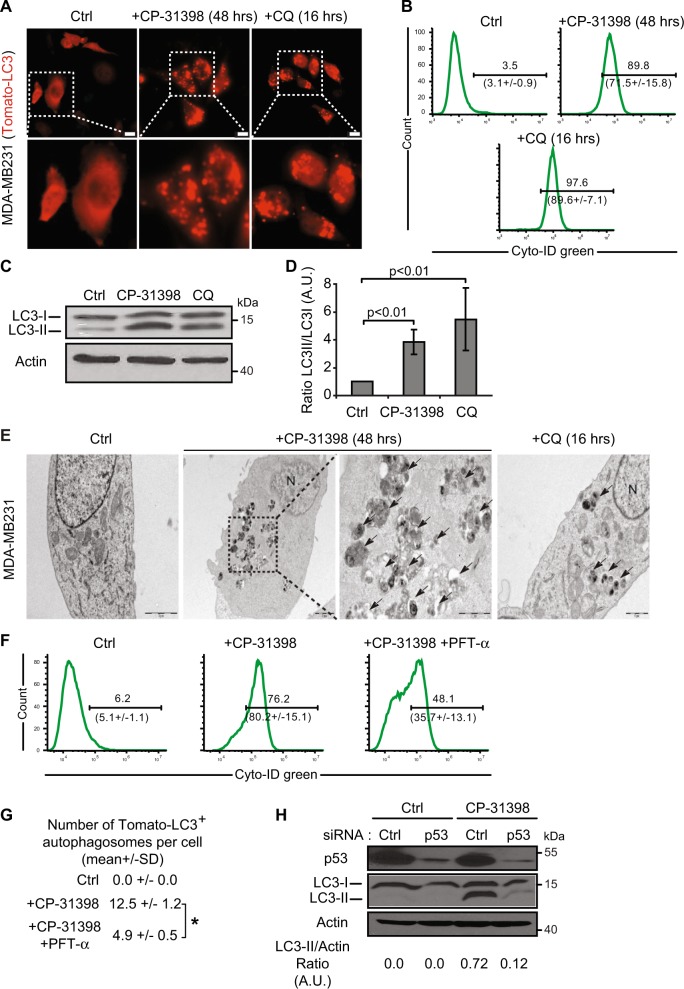
The reactivation of p53 by CP-31398 triggers the formation of autophagosomes. **a** Tomato-LC3 fusion protein is mostly diffuse throughout the cytoplasm of untreated cells, while punctate staining, representing autophagosomes, are observed when MDA-MB231 cells are treated with CP-31398, or chloroquine (CQ; used as positive control). Scale bars: 20 μm. **b** CP-31398-induced autophagy was quantified by flow cytometry using CYTO-ID green, which exhibiting bright fluorescence upon incorporation into pre-autophagosomes, autophagosomes, and autophagolysosomes. The upper number indicates the percentage positive cells, and the lower number the mean ± s.d. of three independent experiments. **c**, **d** Expression of the phosphatidylethanolamine-conjugated form of LC3 (or LC3-II) (**c**) and the LC3-II/LC3-I ratio (**d**) increase following CP-31398 (48 h) treatment of MDA-MB231 cells. **e** Effect of CP-31398 or chloroquine (CQ) on autophagosomes formation observed by electron microscopy. N nucleus, black arrows autophagosomes. **f**, **g** The inhibitor of p53 transcriptional activity PFT-α decreases CP-31398-induced autophagosomes formation, measured by flow cytometry using CYTO-ID green (**f**) or by counting the number of LC3-Tomato + puncta per cell in fluorescence microscopy pictures (**g**). *P < 0.05 (unpaired two-tailed Student's *t* test). **h** The inhibition of p53 expression using siRNA strongly decrease CP-31398-induced LC3 lipidation (LC3-II) measured by western blot. The normalized LC3-II/Actin ratio was calculated by densitometry and display. Data are representative of three independent experiments (**a**, **c**, **e**, **h**) or are the mean ± s.d. of three independent experiments (**b**, **d**, **f**, **g**)

Since our results demonstrated that CP-31398 could initiate autophagy, we used different approaches to evaluate the presence of autophagosomes in the treated cells. Autophagy was measured by following the distribution pattern of microtubule-associated proteins light chain 3 (MAP-LC3; referred as LC3 thereafter) fused with a Tomato fluorescent tag (Tomato-LC3). Tomato-LC3 displayed clear punctate staining in CP-31398-treated MDA-MB231 cells, suggesting the presence of autophagosomes, as in cells treated during 16 h with chloroquine (CQ) that impairs autophagosomes fusion with lysosomes, allowing LC3 accumulation in autophagosomes even under normal condition and revealing the basal autophagic flux^40^ (Fig. 4a and Supplementary figure S3A). Similar results were obtained with a flow cytometry-based monitoring of autophagy using CYTO-ID green, a dye that allow for minimal staining of lysosomes while exhibiting bright fluorescence upon incorporation into autophagosomes and autophagolysosomes^41^ (Fig. 4b). This was also reflected by the increased expression of phosphatidylethanolamine (PE)-conjugated LC3 (or LC3-II) following CP-31398 treatment (Fig. 4c, d). Similar results were also obtained with SKBR3 and T47D cells (Supplementary figure S4A). Finally, transmission electron microscopy analysis also revealed the presence of autophagosomes in CP-31398 treated MDA-MB231 cells (Fig. 4e and supplementary figure S3B) further demonstrating that CP-31398 induces autophagy, most likely through the reactivation of p53 transcriptional activity. To examine this last point, MDA-MB231 cells were treated with CP-31398 in combination with PFT-α before examining autophagosomes formation. As expected, PFT-α decreases the percentage CYTO-ID green positive cells and the number of Tomato-LC3^+^ autophagosomes per cell after CP-31398 treatment (Fig. 4f, g). Similarly, the inhibition of mutant p53 expression using siRNA strongly decrease LC3-II expression in MDA-MB231 cells after CP-31398 treatment (Fig. 4h). Of note, in wt p53 MCF7 breast cancer cells, CP-31398 has a minor effect on LC3-II induction, compared to CQ treatment (Supplementary figure S4B). Together, these data demonstrate that the reactivation of p53 transcriptional activity in MDA-MB231 cells using CP-31398 triggers the formation of autophagosomes.

### Autophagy is responsible for the CP-31398-dependent potentiation of p53-mutated breast tumor cell lysis by NK cells

**Fig. 5:**
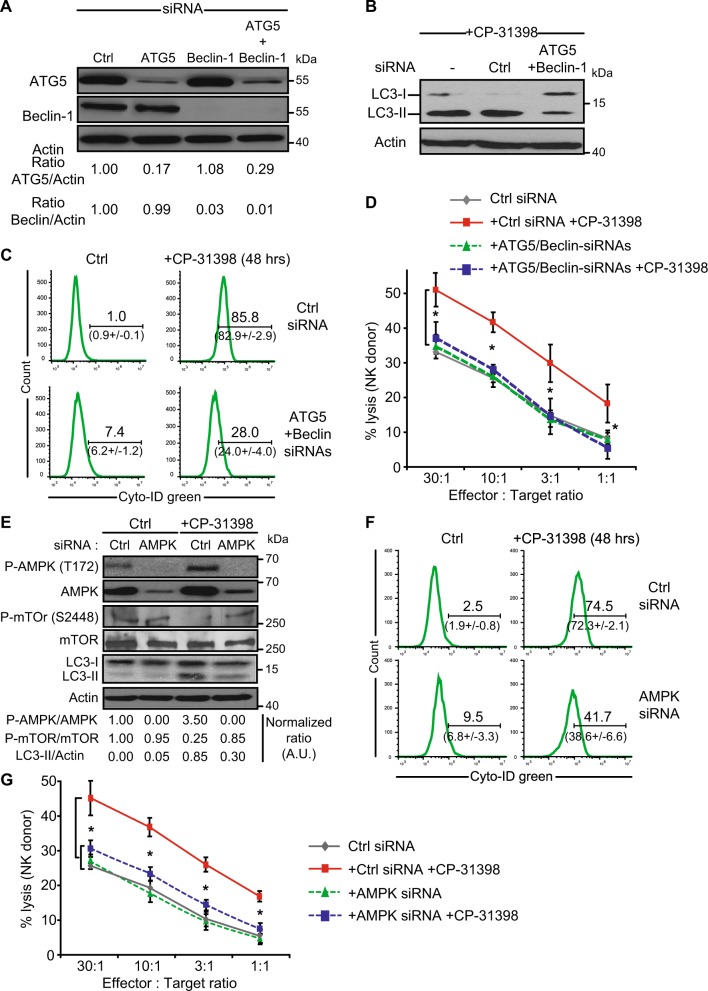
CP-31398-dependent induction of autophagy is crucial for the potentiation of NK-mediated lysis. **a** MDA-MB231 cells were transfected with siRNAs to inhibit two key proteins involved in autophagy, Atg5 and Beclin-1. The normalized ratio of Atg5 or Beclin-1/Actin were calculated by densitometry and display. **b**, **c** The double knockdown of Atg5 and Beclin-1 decreases CP-31398-induced autophagosomes formation, measured by evaluating the phosphatidylethanolamine conjugation of LC3 (or LC3-II) (**b**) or by flow cytometry using CYTO-ID green (**c**). Numbers indicate the percentage positive cells in the displayed representative flow cytometry histograms and the mean ± s.d. from three independent experiments. **d** The inhibition of autophagy using Atg5 and Beclin-1 siRNAs inhibits CP-31398-induced increase of MDA-MB231 lysis by NK cells. **e** MDA-MB231 cells transfection with AMPK siRNAs decreases CP-31398-induced AMPK phosphorylation (T172) and m-TOR dephosphylation (S2448), leading to a decrease of LC3 lipidation (LC3-II). **f** The knockdown of AMPK decreases CP-31398-induced autophagosomes formation, measured by flow cytometry using CYTO-ID green. **g** The inhibition of autophagy using AMPK siRNAs strongly decrease CP-31398-induced potentiation of MDA-MB231 lysis by NK cells. ^51^Cr release assay using NK cells isolated from a healthy donor (**d**–**g**) co-cultured with target cells at different E:T ratios are shown. Data are representative of at least three independent experiments (**a**, **b**, **e**) or are the mean ± s.d. of at least three independent experiments (**c**, **d**, **f**, **g**). The p values (**d**, **g**) were determined using one-way ANOVA. *P < 0.05

We next aimed to validate that CP-31398-induced autophagy is responsible for the observed increased of tumor cell susceptibility to NK-mediated killing. For this purpose, we inhibited the expression of ATG5 (involved in autophagosomal membrane elongation) and Beclin-1 (involved in the regulation of autophagy through its interaction with Bcl-2)^42^ in MDA-MB231 cells using siRNAs, alone or in combination (Fig. 5a). As expected, the combination of ATG5 and Beclin-1 siRNAs strongly reduces CP-31398-induced LC3-II expression and autophagosome formation, measured using CYTO-ID green staining (Fig. 5b, c). Importantly, the use of ATG5 and Beclin-1 siRNAs totally abrogates the CP-31398-dependent potentiation of MDA-MB231 lysis by NK cells (Fig. 5d). Similarly, the use of AMPK siRNAs to interfere with the AMPK-mTOR pathway inhibits CP-31398-induced AMPK phosphorylation, mTOR dephosphorylation and LC3-II induction (Fig. 5e). AMPK siRNAs also inhibit autophagy induction, measured by flow cytometry (Fig. 5f) and prevent CP-31398-mediated potentiation of the NK-mediated lysis (Fig. 5g). Together, these results clearly demonstrate that the induction of autophagy by CP-31398 is responsible for the observed potentiation of p53-mutated tumor cells lysis by NK cells.

### CP-31398 triggers a p62 and NBR1-independent selective sequestration of anti-apoptotic proteins in autophagosomes

**Fig. 6:**
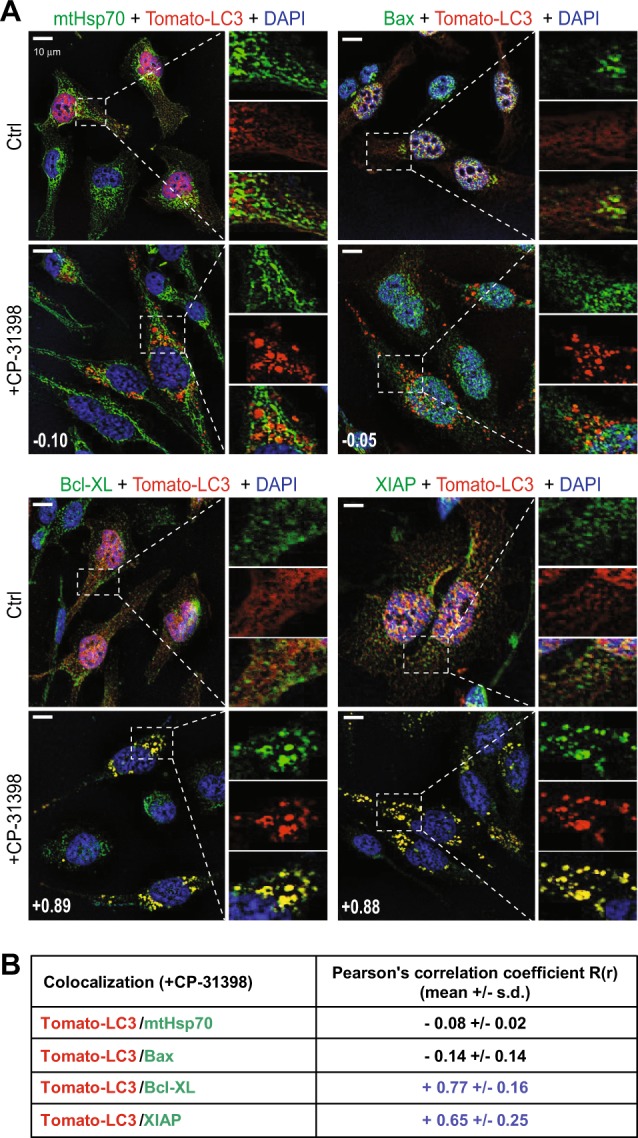
CP-31398 triggers the sequestration of Bcl-X_L_ and XIAP in autophagosomes. **a** CP-31398-induced Tomato-LC3^+^ autophagosomes contains the anti-apoptotic proteins Bcl-X_L_ and XIAP, but not the mitochondrial marker mtHsp70 and the pro-apoptotic protein Bax. Scale bars: 20 μm. Data are representative of three independent fluorescence microscopy experiments. **b** The colocalization between CP-31398-induced Tomato-LC3^+^ autophagosomes and the indicated proteins (stained in green) was measured and displayed as a pearson’s correlation coefficient *R*(*r*) (also indicated as a number in (**a**). The data (mean ± s.d.) obtained from 20 images containing at least five cells from three independent experiments are shown. Bcl-X_L_ and XIAP staining (*R*(*r*) > 0.6) is strongly associated with Tomato-LC3^+^ autophagosomes

We then aimed to investigate the mechanisms linking the induction of autophagy by CP-31398 and the potentiation of p53-mutated breast tumor cells lysis by NK cells. The killing of MDA-MB231 by NK cells is mostly dependent on the PFN/GzmB pathway, as shown by the almost complete inhibition of NK-mediated lysis in presence of concanamycin A (CMA) (a Ca^2+^ chelator that inhibits cytotoxic granules exocytosis) (Supplementary figure S5). We thus hypothesized that CP-31398-induced autophagy improves the lysis of tumor cells through the sequestration/degradation of key negative regulators of the GzmB apoptotic pathway. Fluorescence microscopy results demonstrate that the mitochondrial marker mtHsp70, the pro-apoptotic protein Bax (used as controls) or the anti-apoptotic proteins Mcl-1 and cIAP-2 were not present in CP-31398-induced Tomato-LC3^+^ autophagosomes. On the opposite, Bcl-X_L_ and XIAP were strongly associated with CP-31398-induced autophagosomes. Regarding Bcl-2, cIAP-1 and survivin, their internalization in autophagosomes seems to be less evident (Fig. 6a and Supplementary figure S6A). To further quantify these results, we measured the colocalization between CP-31398-induced Tomato-LC3^+^ autophagosomes and the protein of interest, displayed as a pearson’s correlation coefficient R(r). The data confirmed that CP-31398-induced Tomato-LC3^+^ autophagosomes mostly contains Bcl-X_L_ and XIAP (*R*(*r*) > 0.5) while Bcl-2, cIAP-1 and survivin can be internalized in autophagosomes but at a lower level (*R*(*r*) < 0.5) (Fig. 6b and Supplementary figure S6B). Importantly, this Bcl-X_L_ and XIAP internalization in autophagosomes in response to CP-31398 was greatly impaired in p53 siRNAs transfected MDA-MB231 cells, which decreases the number of CP-31398-induced Tomato-LC3^+^ autophagosomes (Supplementary figure S7A–C). Of note, Bcl-X_L_ and XIAP were mostly not internalized in CQ-induced autophagosomes and CQ treatment was not associated with a potentiatition of MDA-MB231 cell killing by NK cells (Supplementary figure S8A, B), further demonstrating the importance of a selective Bcl-X_L_ and XIAP internalization in autophagosomes for the CP-31398 effect on NK-mediated killing. Selectivity in autophagy is conferred by cargo receptor proteins^43^, such as Sequestosome-1 (p62/SQSTM1) and Neighbor of BRCA1 gene 1 (NBR1). Using fluorescence microscopy, we observed that both p62 and NBR1 relocalize to CP-31398-induced autophagosomes (Supplementary figure S9A, B). However, immunoprecipitation experiments showed that neither p62 nor NBR1 interact with Bcl-X_L_ or XIAP in response to CP-31398, while these proteins cargo interacts with LC3-II, used as a positive control (Supplementary figure S9C, D). Together, these data demonstrate that p53-reactivation using CP-31398 induces a p62 and NBR1-independent selective autophagy leading to Bcl-X_L_ and XIAP (and probably other anti-apoptotic proteins) internalization in autophagosomes.

### CP-31398-induced autophagy is blocked, interfering with the degradation of the internalized anti-apoptotic proteins

**Fig. 7:**
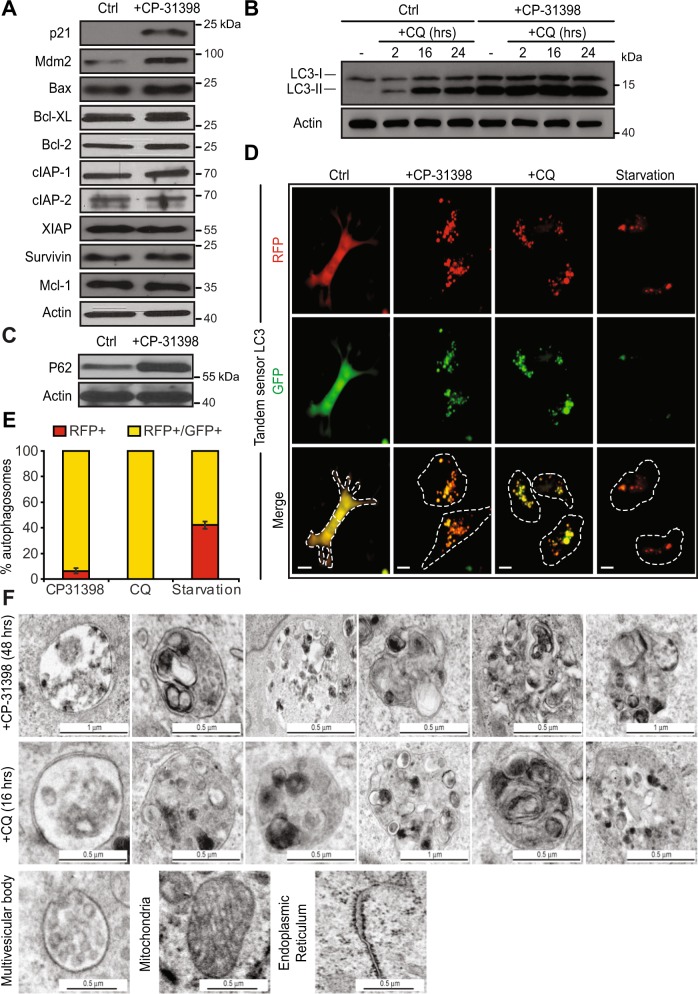
CP-31398-induced autophagic flux is blocked at the autophagosome-lysosome fusion step. **a** CP-31398 treatment (48 h) increases p21 and Mdm2 expression by MDA-MB231 cells, does not alter Bax level (used as control) and does not induce the degradation of Bcl-X_L_, XIAP, Bcl-2, Mcl-1, cIAP-1, IAP-2, or survivin. **b** The differences in the amount of LC3-II between samples in the presence and absence of chloroquine (CQ), representing the amount of LC3 that is degraded into autolysosomes, and allowing the measurement of the autophagic flux is represented. No additional LC3-II accumulation is observed when CP-31398 (48 h) is used in combination with CQ (2–24 h), showing that the autophagic flux induced by CP-31398 is blocked at the autophagosome-lysosome fusion step. **c** P62 expression level increases following 48 h treatment with CP-31398. **d**, **e** MDA-MB231 cells were transduced with LC3 fusion protein tagged with acid‑sensitive eGFP and acid‑insensitive RFP to monitor the specific loss of eGFP fluorescence upon acidification of the RFP^+^ autophagosome following lysosomal fusion. Serum starvation induces autophagy with the loss of eGFP signal reflecting the fusion of autophagosomes with lysosomes. Fluorescence is seen from both eGFP and RFP channels after CP-31398 treatment, similarly to CQ that blocks autophagy through neutralization of lysosomal pH, demonstrating that CP-31398-induced autophagy flux is blocked at the autophagosomes/lysosomes fusion step. Dashed lines: plasma membrane. Scale bars: 10 μm (**c**). The percentage RFP^+^ or RFP^+^/GFP^+^ autophagosomes was evaluated in 100 cells after the indicated treatment (**d**). **f** Representative electron microscopy images of the autophagosomes containing undigested material presents in CP-31398 or chloroquine (CQ)-treated cells. Representative images of multivesicular bodies, mitochondria and endoplasmic reticulum are displayed as control. Data are representative of three independent experiments (**a**–**d**, **f**) or are the mean ± s.d. of three independent experiments (**e**)

We then studied whether CP-31398-induced autophagy leads to the degradation of the internalized anti-apoptotic proteins. Western blot analysis of CP-31398-treated MDA-MB231 cells surprisingly did not reveal any change in Bcl-X_L_, XIAP, Bcl-2, cIAP-1 or survivin amount (or in Bax, Mcl-1, or cIAP-2 protein level used as control) (Fig. 7a). We thus hypothesized that the CP-31398-induced autophagic flux (defined as the autophagic degradation activity^44^) is blocked and that the absence of degradation could be due to the absence of fusion between autophagosomes and lysosomes. To validate this hypothesis, we performed a LC3 turnover assay using western blot and CQ. Indeed, in response to CQ, which inhibits lysosome acidification and autophagosome-lysosome fusion, LC3-II accumulates even under normal conditions because the turnover/degradation of LC3-II by basal autophagy is blocked. Accordingly, the differences for LC3-II level between samples in the presence and absence of CQ represent the amount of LC3 that is degraded into autolysosomes, allowing the measurement of the autophagic flux^40^. As expected, CQ alone induces a time dependent LC3-II accumulation in MDA-MB231 cells, suggesting that basal autophagy is functional. However, when CP-31398 was combined with CQ, no additional LC3-II accumulation was observed as compare to CP-31398 alone (Fig. 7b). These results suggest that the autophagy induced by CP-31398 is blocked at the autophagosome-lysosome fusion step, which is also reflected by the accumulation of p62 in response to CP-31398 treatment (Fig. 7c). To further validate this point, we used a microscopy approach based on a LC3 fusion protein tagged with acid‑sensitive eGFP and acid‑insensitive RFP. This fusion protein allows monitoring the change from an autophagosome (neutral pH) to an autolysosome (acidic pH) by visualizing the specific loss of the GFP fluorescence upon acidification of the autophagosome following lysosomal fusion. As shown in Fig. 7d, e, serum starvation (positive control) induces autophagy with the loss of eGFP signal reflecting the fusion of autophagosomes with lysosomes. On the opposite, CQ blocks autophagy through neutralization of lysosomal pH and as a result, fluorescence was observed from both eGFP and RFP channels. Interestingly, fluorescence was also seen from both eGFP and RFP channels during CP-31398-triggered autophagy, suggesting a block. Finally, CP-31398-induced autophagosomes observed by electron microscopy are very similar to CQ-triggered autophagosomes, where autophagosome-lysosome fusion is blocked, with a frequent irregular shape and large amount of undigested content (Fig. 7f). Together, these data demonstrate that CP-31398 both induces autophagy and blocks the autophagosomes/lysosomes fusion step, which interferes with the degradation of the anti-apoptotic proteins sequestrated in autophagosomes.

### CP-31398-induced sequestration of anti-apoptotic proteins in autophagosomes correlates with an increase of granzyme B-induced cell death

**Fig. 8:**
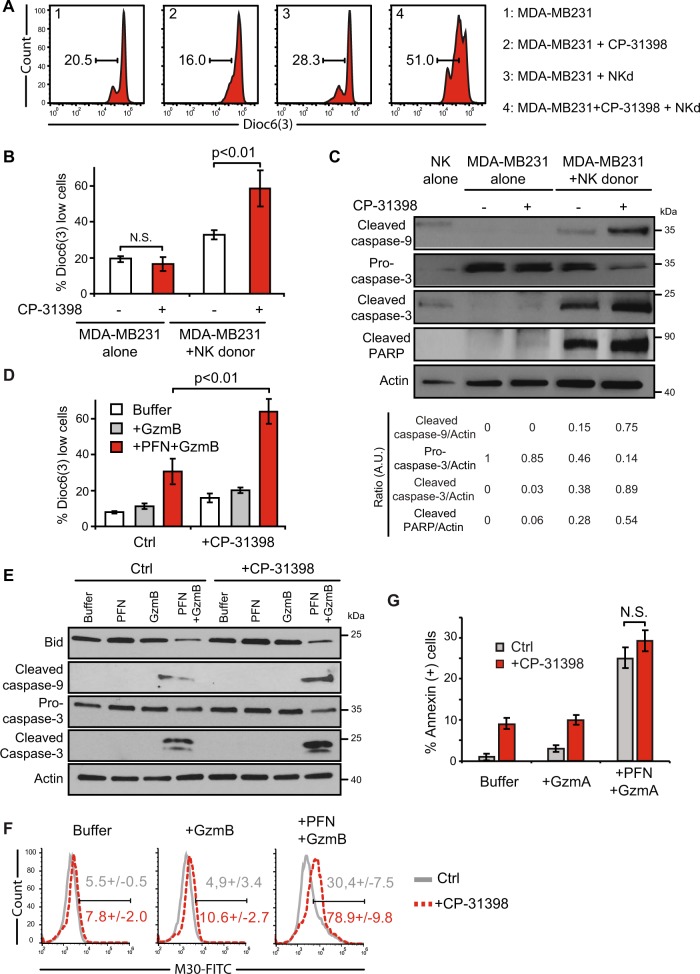
CP-31398-induced sequestration of several anti-apoptotic proteins in autophagosomes potentiates granzyme B-induced MOMP and caspase-3 cleavage. **a**, **b** CP-31398 improves mitochondrial outer membrane permeabilization (MOMP) measured by Dioc6(3) staining in MDA-MB231 cells incubated with NK cells isolated from a healthy donor (NKd) at the E:T ratio of 5:1. **c** CP-31398 increases caspase-3 cleavage in MDA-MB231 cells incubated with NK cells isolated from a healthy donor (NKd) at the E:T ratio of 3:1. The normalized ratio of full length or cleaved caspase-3, -9 or PARP/Actin was calculated by densitometry analysis. **d**, **e** CP-31398 increases MOMP measured by Dioc6(3) staining (**c**) and caspases-9 and -3 cleavage (**d**) in MDA-MB231 cells treated 30 min with PFN/GzmB. **f** CP-31398 increases PFN/GzmB-induced apoptosis, measured by flow cytometry using M30 mAb staining (which recognizes a cytokeratin-18 epitope, revealed after effector caspase cleavage). Representative flow cytometry histograms are shown. Numbers indicate the percentage M30 positive cells (mean ± s.d. from three independent experiments). **g** PFN/GzmA-induced cell death, measured by Annexin-V staining, is unaltered following CP-31398 treatment. Data are representative of three independent experiments (**a**, **c**, **e**) or are the mean ± s.d. of three independent experiments (**b**, **d**, **g**). The *p* values (**b**, **d**, **g**) were determined by unpaired two-tailed Student's *t* test. *NS* non-significant

We finally examined whether the sequestration without degradation of Bcl-X_L_ and XIAP in CP-31398-induced autophagosomes leads to an increase of the well-known apoptotic signaling triggered by PFN/GzmB through the mitochondrial and effector caspases pathway^45^, which would explain the observed potentiation of NK-mediated lysis. We first evaluated NK cell-induced MOMP in MDA-MB231 cells ± CP-31398, using DioC6(3). Flow cytometry analysis revealed that CP-31398 potentiates MOMP induced by NK cells (Fig. 8a, b). Furthermore, CP-31398 potentiated the cleavage of caspase-9 and -3 and PARP in response to NK cells (Fig. 8c). Similarly, CP-31398 increased MOMP and caspase-9 and -3 activation induced by PFN/GzmB (Fig. 8d, e). Importantly, Bid cleavage in response to PFN/GzmB was not affected by CP-31398, further demonstrating that CP-31398 treatment only affects downstream events. Consequently, CP-31398 was associated with a potentiation of PFN/GzmB-induced apoptosis (Fig. 8f). Of note, granzyme A (GzmA)-induced cell death, which rely on ROS and on the SET complex, but is caspases, Bax/Bak, Bcl-2/Bcl-X_L_ and cytochrome c independent^46–49^, was not increased following CP-31398 treatment (Fig. 8g). Together, these results indicate that the sequestration in CP-31398-induced autophagosomes of several anti-apoptotic proteins, including Bcl-X_L_ and XIAP, which normally prevent GzmB-induced MOMP and effector caspases cleavage, potentiates GzmB- and NK-dependent apoptosis of p53-mutated cells, without the need of degradation in autophagosomes.

## Discussion

Here, we demonstrated that the pharmalogical reactivation of a wt-like p53 transcriptional function in p53-mutated breast cancer cells using the small molecule CP-31398 increases their susceptibility to NK cell-mediated lysis by an autophagy-dependent mechanism. In particular, we found that CP-31398 treatment induces the expression of Sestrin-1 and Sestrin-2, two known p53 targets^19^, which in turn facilitates AMPK phosphorylation and consequently leads to mTOR inhibition. In parallel, we observed that CP-31398 strongly increases ULK1 expression in a p53-dependent manner. Consequently, the inhibition of mTOR activity and the increase ULK1 availability, leads to the formation of autophagosomes in CP-31398 treated cells, as confirmed by the effect of siRNA-based AMPK inhibition on CP-31398-dependent induction of autophagy. It was also conceivable that some of the observed effect of CP-31398 on autophagy and NK cell-mediated lysis could be independent of p53 transcriptional activity reactivation, as previously described^37,50,51^. However, we excluded this possibility since the knockdown of mutant p53 expression with siRNA or the inhibition of p53 transcriptional activity with PFT-α block both the CP-31398-dependent autophagosomes formation in p53-mutated cells and the potentiation of their killing by NK cells. Of note, a previous study demonstrated that CP-31398 could unspecifically activate autophagy in wt p53 pancreatic tumor cells^52^. However, using the wt p53 MCF7 breast cancer cells, we only observed a minor LC3-II induction in response to CP-31398 in comparison to p53-mutated cells, which seems to be insufficient to increase MCF7 lysis by NK cells and suggesting that the induction of autophagy using this small compound is mostly effective in p53-mutated cells. Several reports have also shown that CP-31398 can induce oxidative stress^53,54^ which in turn could induce autophagy^55,56^. However, we did not observed any significant ROS production following CP-31398 treatment in our model.

Importantly, our findings also clearly demonstrate that CP-31398-mediated autophagosome formation in p53-mutated breast cancer cells is the main event involved in the potentiation of their lysis by GzmB and NK cells. This phenomenon is linked to the autophagosomal sequestration of anti-apoptotic proteins, especially Bcl-X_L_ and XIAP, in CP-31398-induced autophagosomes, and is associated with an increased MOMP and caspase-9 and -3 cleavage in response to both GzmB and NK cells. Indeed, the sequestration of anti-apoptotic proteins like Bcl-X_L_ and XIAP probably prevents their inhibitory interaction with pro-apoptotic partners, facilitating apoptosis induction by additional external stimuli that trigger MOMP and effector caspase activation, such as GzmB and NK cells, but not GzmA which induces cell death in a caspases and Bcl-2 family members-independent manner. Interestingly, we also observed that Bcl-2, cIAP-1 and survivin internalization seems possible at a lower extend, but not cIAP-2, Mcl-1. This apparent selectivity in CP-31398-induced autophagy seems to be independent from p62 and NBR1 cargo receptors, classically involved in different selective autophagy pathways^43^. Importantly, we also observed that autophagosomes accumulation in response to CQ is not associated with a Bcl-X_L_ and XIAP sequestration within these structures and consequently with an increase of NK cell-dependent cytotoxicity. Thus, the selective addressing of Bcl-X_L_ and XIAP to autophagosomes is also an important feature to explain the effect of CP-31398 on NK/GzmB-mediated lysis. This selectivity could also explain apparent contradictory observations regarding the role of autophagy in NK cell-mediated lysis. Indeed, several reports have shown that autophagy induction can protect tumor cells against NK cell-mediated killing^57–60^. Thus, the selectivity in the cargo internalized in autophagosomes in response to specific stimuli, could either protect or sensitize cells to external cell death inducers, such as NK cells, even if this point clearly needs further experimental validation.

During this study, we also demonstrated that CP-31398-induced autophagy was not fully functional and that the autophagosomes-lysosomes fusion process was most likely dysfunctional, explaining the absence of degradation of the internalized anti-apoptotic proteins. In this regard, previous studies have shown that the fusion of autophagosomes with lysosomes requires the conventional cellular fusion machinery involving proteins such as syntaxin^61^. Interestingly, our microarray results indicate that several syntaxin expression (including *syntaxin-2*, -*7*, -*8*, -*10*) was downregulated following CP-31398 treatment (data not shown). Similarly, we observed a strong downregulation of *CLN6* expression (data not shown) following CP-31398 treatment. CLN6 is an endoplasmic-associated protein and despite its location, contributes to lysosomal function by an unknown mechanism. Moreover, CLN6 mutations/deficiency are associated to lysosomal function disorders and to a disruption of the autophagy-lysosome degradation pathway^62^. Thus, the CP-31398-mediated blockage of autophagy might result from several alterations of the autophagosomes-lysosomes fusion machinery, potentially involving syntaxins and CLN6. However, this point clearly needs further validation, in particular to determine whether this block is p53-dependent or a CP-31398 “side effect”.

In conclusion, this study emphasizes that the use of p53-reactivating molecules can potentiate the lysis of p53-mutated breast cancer cells by NK cells though autophagy induction. Furthermore, these findings can probably be extended to other tumor types with high p53 mutation rate such as lung cancer and to CTL-mediated lysis because both NK cell and CTL mainly used the same PFN/GzmB pathway to kill their target cells. Thus, our results could define a new way to increase cytotoxic lymphocyte-mediated lysis of p53-mutated cancer cells, with potential application in immunotherapeutic approaches. Indeed, the development of effective p53-reactivating small molecules, such as APR-246^63^, could open the way of combined treatments between these molecules and immunotherapeutic approaches such as immune checkpoint blockade or CAR-T cells treatment. These points will be analyzed in future in vivo studies.

## Supplementary information

Supplementary Figures
