# Supplementary material for: The pharmalogical reactivation of p53 function improves breast tumor cell lysis by granzyme B and NK cells through induction of autophagy
Source: Cell Death Dis. 2019 Sep 20;10(10):695. doi: 10.1038/s41419-019-1950-1 (PMC6754511; doi:10.1038/s41419-019-1950-1)
Supplement: Supplementary file 1 — Supplementary Figures [file 41419_2019_1950_MOESM1_ESM.pdf]

Figure S1

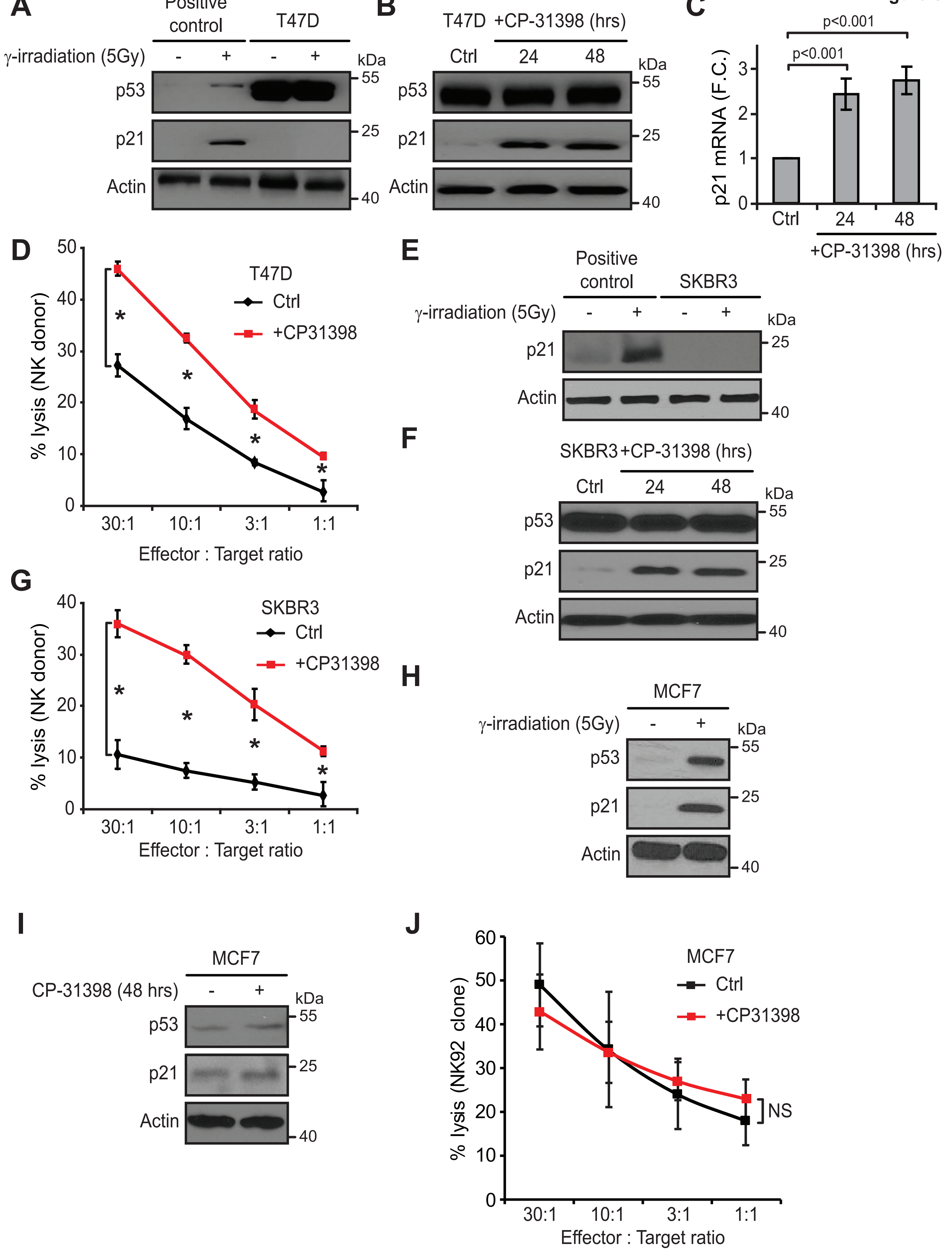
A
Positive control
T47D
γ-irradiation (5Gy)
- + - +
kDa
p53
p21
Actin
55
25
40
B
T47D +CP-31398 (hrs)
Ctrl 24 48
kDa
p53
p21
Actin
55
25
40
C
p<0.001
p<0.001
p21 mRNA (F.C.)
Ctrl 24 48
+CP-31398 (hrs)
D
T47D
Ctrl
+CP31398
% lysis (NK donor)
30:1 10:1 3:1 1:1
Effector : Target ratio
E
Positive control
SKBR3
γ-irradiation (5Gy)
- + - +
kDa
p21
Actin
25
40
F
SKBR3+CP-31398 (hrs)
Ctrl 24 48
kDa
p53
p21
Actin
55
25
40
G
SKBR3
Ctrl
+CP31398
% lysis (NK donor)
30:1 10:1 3:1 1:1
Effector : Target ratio
H
MCF7
γ-irradiation (5Gy)
- +
kDa
p53
p21
Actin
55
25
40
I
MCF7
CP-31398 (48 hrs)
- +
kDa
p53
p21
Actin
55
25
40
J
MCF7
Ctrl
+CP31398
% lysis (NK92 clone)
NS
30:1 10:1 3:1 1:1
Effector : Target ratio

Figure S1. The reactivation of p53 transcriptional activity by CP-31398 increases T47D and SKBR3 breast cancer cell susceptibility to NK-mediated lysis

(**A,E)** T47D and SKBR3 cells express high level of mutated p53 ($p53^{L194F}$ and $p53^{R175H}$ respectively) lacking transcriptional activity as shown by the absence of p53-dependent transactivation of its target gene p21 even after γ-irradiation. **(B-C,F)** 24-48 hrs treatment with CP-31398 reactivates p53 transcriptional activity in T47D and SKBR3 cells as shown by induction of p21 expression at mRNA (C) and protein (B-F) levels. **(D,G)** CP-31398 treatment (48 hrs) increases T47D and SKBR3 cells susceptibility to NK-mediated lysis. ^{51}Cr release assays using NK cells isolated from a healthy donor co-cultured with target cells at different E:T ratios are shown. **(H-J)** MCF7 cells express a transcriptionally active wt p53 as shown by transactivation of its target gene p21 after γ-irradiation (H). CP-31398 did not induce p21 expression in these cells (I) neither potentiate their lysis by NK cells (NK92) (J). Data are representative of three independent experiments (A-B, E-F, H-I) or are the mean ± s.d. of three (D, G, J) or five (C) independent experiments. The p values (C-D, G, J) were determined by unpaired two-tailed Student t test. NS: non-significant. $*P < 0.05$.

Figure S2

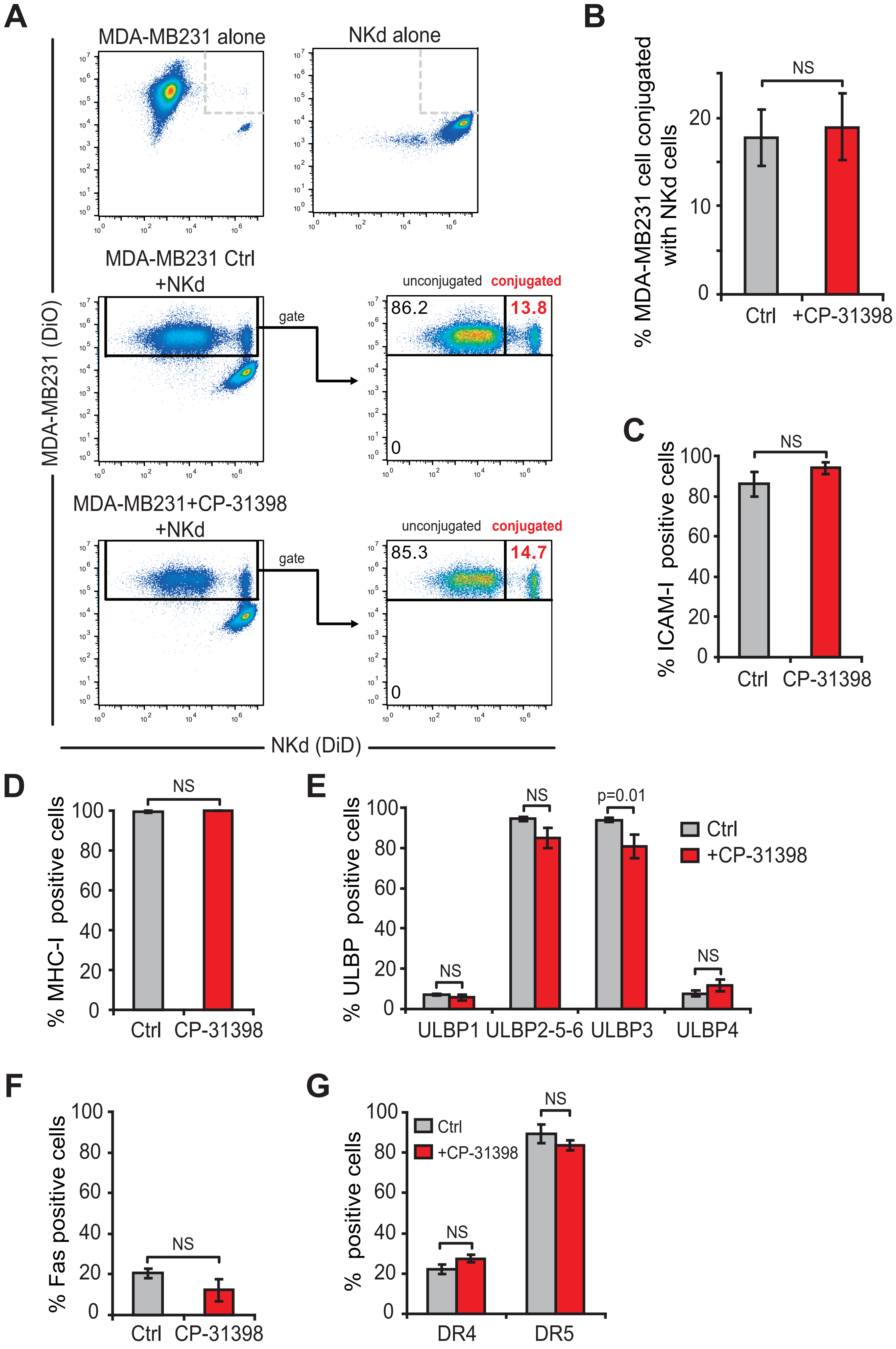

A
MDA-MB231 alone
NKd alone
MDA-MB231 Ctrl +NKd
gate
unconjugated conjugated
86.2
13.8
0
MDA-MB231+CP-31398 +NKd
gate
unconjugated conjugated
85.3
14.7
0
MDA-MB231 (DiO)
NKd (DiD)
B
% MDA-MB231 cell conjugated with NKd cells
NS
Ctrl
+CP-31398
C
% ICAM-I positive cells
NS
Ctrl
CP-31398
D
% MHC-I positive cells
NS
Ctrl
CP-31398
E
% ULBP positive cells
NS
NS
p=0.01
NS
Ctrl
+CP-31398
ULBP1
ULBP2-5-6
ULBP3
ULBP4
F
% Fas positive cells
NS
Ctrl
CP-31398
G
% positive cells
Ctrl
+CP-31398
NS
NS
DR4
DR5

Figure S2. The reactivation of p53 transcriptional activity by CP-31398 does not improve the formation of conjugates between target and NK cells or increase death receptor expression

(A-B) MDA-MB231 cells ± CP-31398 (48 hrs) stained with the lypophilic dyes DiO were incubated with NKd effector cells, stained with the lypophilic dyes DiD, at the effector:target ratio of 3:1 during 20 min before measuring immune conjugates formation by flow cytometry. Isolated MDA-MB231 and NKd were used as control to identify the two separated populations. The percentage of MDA-MB231 cells conjugated with NKd cells was calculated by gating on target cells and on measuring the percentage of DiO/DiD double positive population representing the immune conjugates, and indicated in red. Representative flow cytometry dot plots (A) and mean ± s.d. of percentage MDA-MB231 cells conjugated with NKd cells (B) from three independent experiments are shown. **(C-G)** ICAM-1, MHC-class I, ULBPs, FAS, DR4 and DR5 expression is not modulated by CP-31398 treatment of MDA-MB231 cells. Data (C-G) are the mean ± s.d. from three independent flow cytometry experiments. The p values (B-G) were determined by unpaired two-tailed Student t test. NS: non-significant.

Figure S3

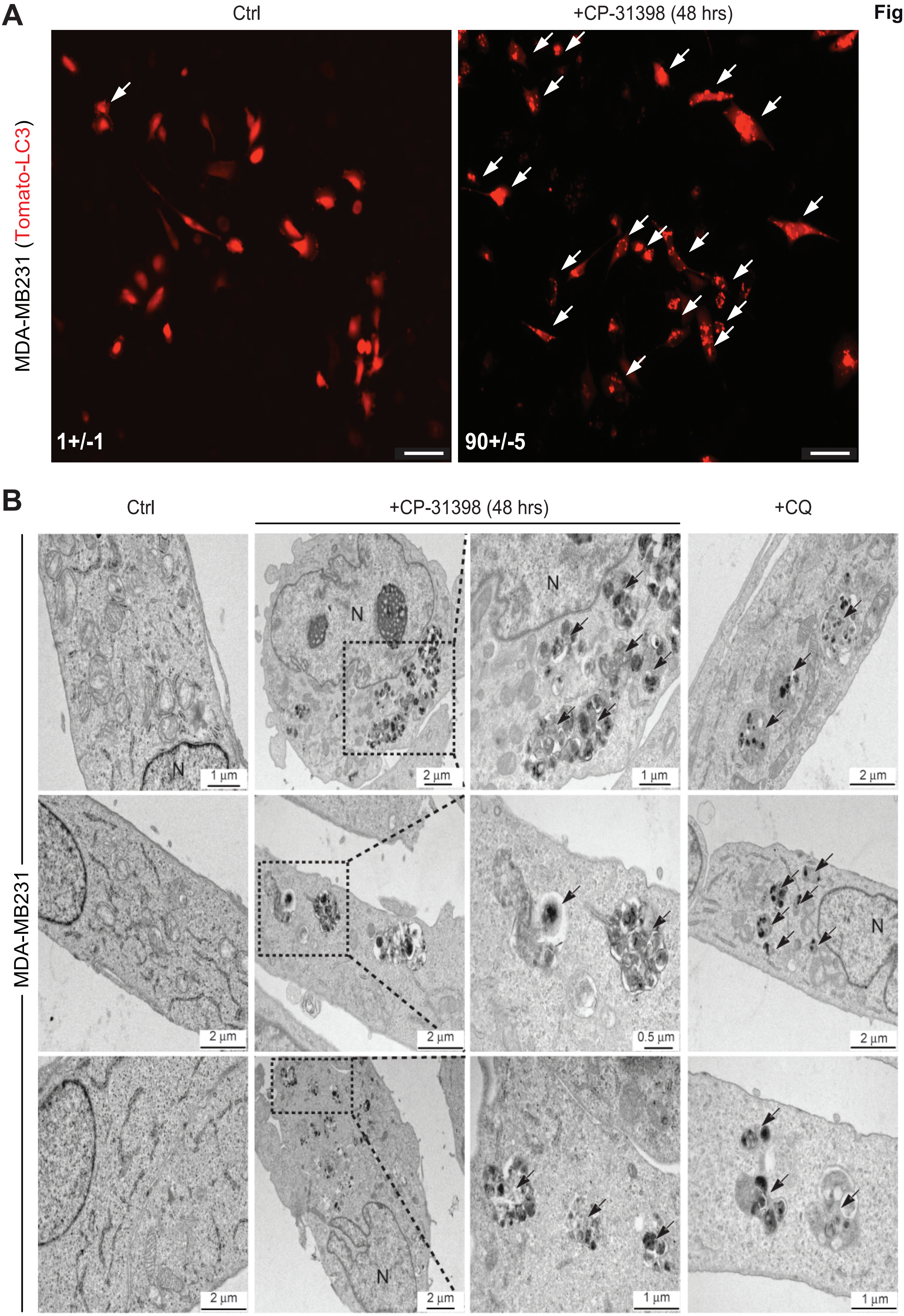
A
Ctrl
+CP-31398 (48 hrs)
MDA-MB231 (Tomato-LC3)
1+/-1
90+/-5
B
Ctrl
+CP-31398 (48 hrs)
+CQ
MDA-MB231
N
1 µm
2 µm
0.5 µm

Figure S3. CP-31398 induces the formation of autophagosomes in MDA-MB231 cells

(A) Tomato-LC3 fusion protein is mostly diffuse throughout the cytoplasm of untreated cells, while punctate staining, representing autophagosomes, are observed when MDA-MB231 cells are treated with CP-31398 (48 hrs). Scale bars: 100 µm. White arrows indicates cells with autophagosomes. Numbers indicate the percentage cell with autophagosomes (mean ± s.d. from three independent experiments). **(B)** Effect of CP-31398 or chloroquine (CQ) on autophagosomes formation observed by electron microscopy. N: nucleus; black arrows: autophagosomes. Data are (A-B) representative of three independent experiments

Figure S4

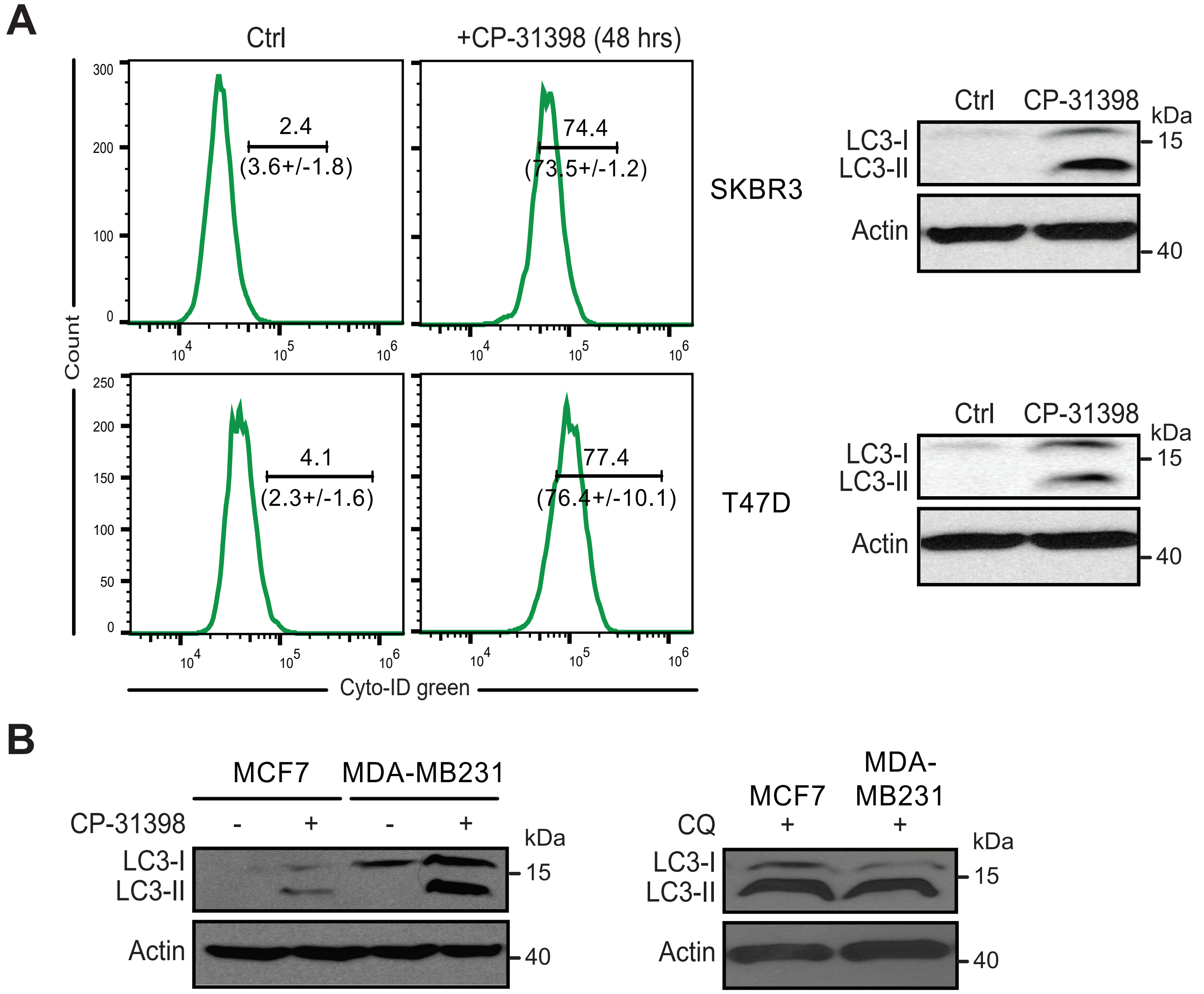
A
Ctrl
+CP-31398 (48 hrs)
Count
Cyto-ID green
2.4
(3.6+/-1.8)
74.4
(73.5+/-1.2)
SKBR3
4.1
(2.3+/-1.6)
77.4
(76.4+/-10.1)
T47D
Ctrl
CP-31398
kDa
LC3-I
LC3-II
Actin
15
40
B
MCF7
MDA-MB231
CP-31398
-
+
-
+
kDa
LC3-I
LC3-II
Actin
15
40
MCF7
MDA-MB231
CQ
+
+
kDa
LC3-I
LC3-II
Actin
15
40

Figure S4. CP-31398 triggers autophagy in SKBR3 and T47D breast tumor cells

(A) CP-31398 induces autophagy in p53-mutated T47D and SKBR3 cells measured by flow cytometry using CYTO-ID green or by western blot to quantify LC3-II. **(B)** Western blot quantification of LC3-II induction in wtp53 MCF7 cells in response to CP-31398 treatment (48 hrs), compared to MDA-MB231 cells (left panel). Chloroquine (CQ) was used as positive control (right panel). Data are representative of three independent experiments (A-B) or are the mean ± s.d. of three independent experiments (A; left panel).

Figure S5

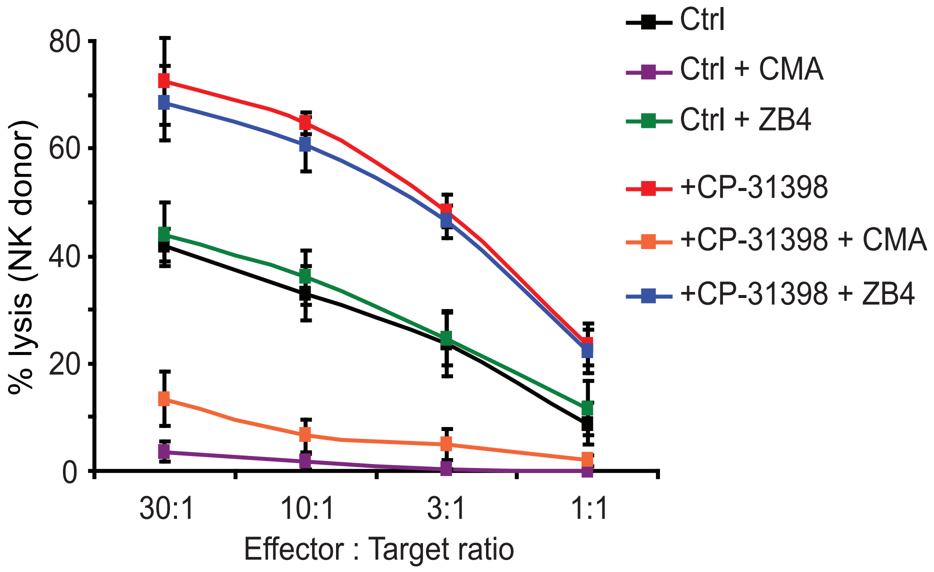

Ctrl
Ctrl + CMA
Ctrl + ZB4
+CP-31398
+CP-31398 + CMA
+CP-31398 + ZB4
% lysis (NK donor)
80
60
40
20
0
30:1
10:1
3:1
1:1
Effector : Target ratio

Figure S5. MDA-MB231 target cells are killed by NK cells through the Perforin/Granzymes pathway

MDA-MB231 lysis by NK cells is dependent on Perforin/Granzymes pathway, indistinctly of CP-31398 treatment, as shown by the almost complete inhibition of NK-mediated lysis in presence of the Ca^{2+} chelator concanamycin A (CMA) (that inhibits cytotoxic granules exocytsosis). The Fas blocking antibody ZB4 was used as control. Data are the mean ± s.d. of two independent ^{51}Cr release assay using NK cells isolated from a healthy donor co-cultured with target cells at different E:T ratios.

Figure S6

A

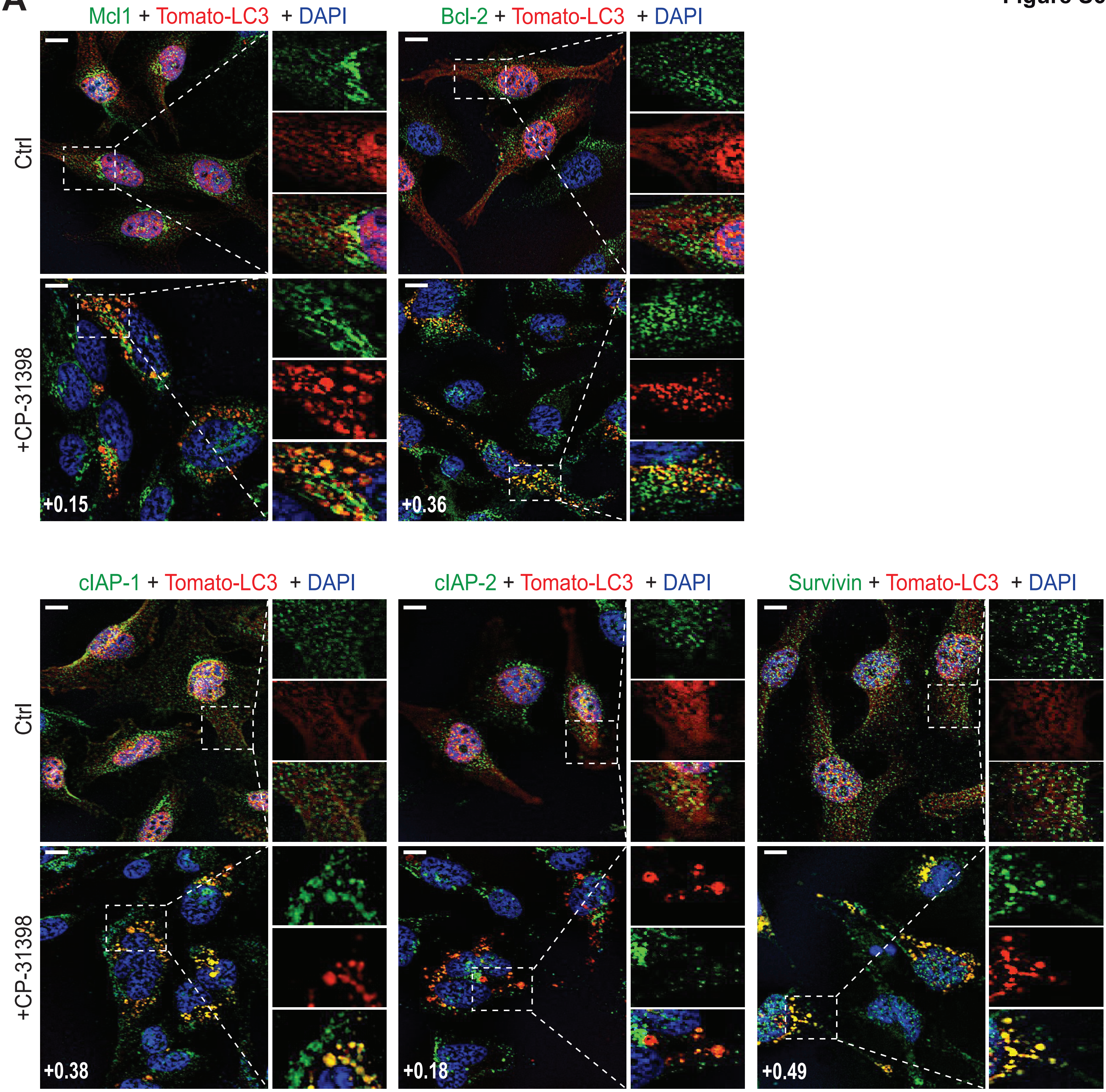

B

Colocalization (+CP-31398)	Pearson's correlation coefficient R(r) (mean +/- s.d.)
Tomato-LC3 /Mcl1	+ 0.10 +/- 0.05
TomatoLC3 /Bcl-2	+ 0.40 +/- 0.10
Tomato-LC3 /cIAP-1	+ 0.33 +/- 0.19
Tomato-LC3 /cIAP-2	+ 0.16 +/- 0.12
Tomato-LC3 /Survivin	+ 0.38+/ - 0.11

Figure S6. CP-31398-induced autophagosomes can contain other anti-apoptotic proteins

(A) CP-31398-induced Tomato-LC3$^+$ autophagosomes do not contain the anti-apoptotic proteins Mcl-1 and cIAP-2, but can contain Bcl-2, cIAP-1 and Survivin at a lower level than Bcl-X_L and XIAP. Scale bars: 20 μm. Data are representative of three independent fluorescence microscopy experiments. **(B)** The colocalization between CP-31398-induced Tomato-LC3$^+$ autophagosomes and the indicated proteins (stained in green) was measured and displayed as a pearson's correlation coefficient R(r) (also indicated as a number in (A). The data (mean ± s.d.) obtained from 20 images from three independent experiments are shown.

Figure S7

A

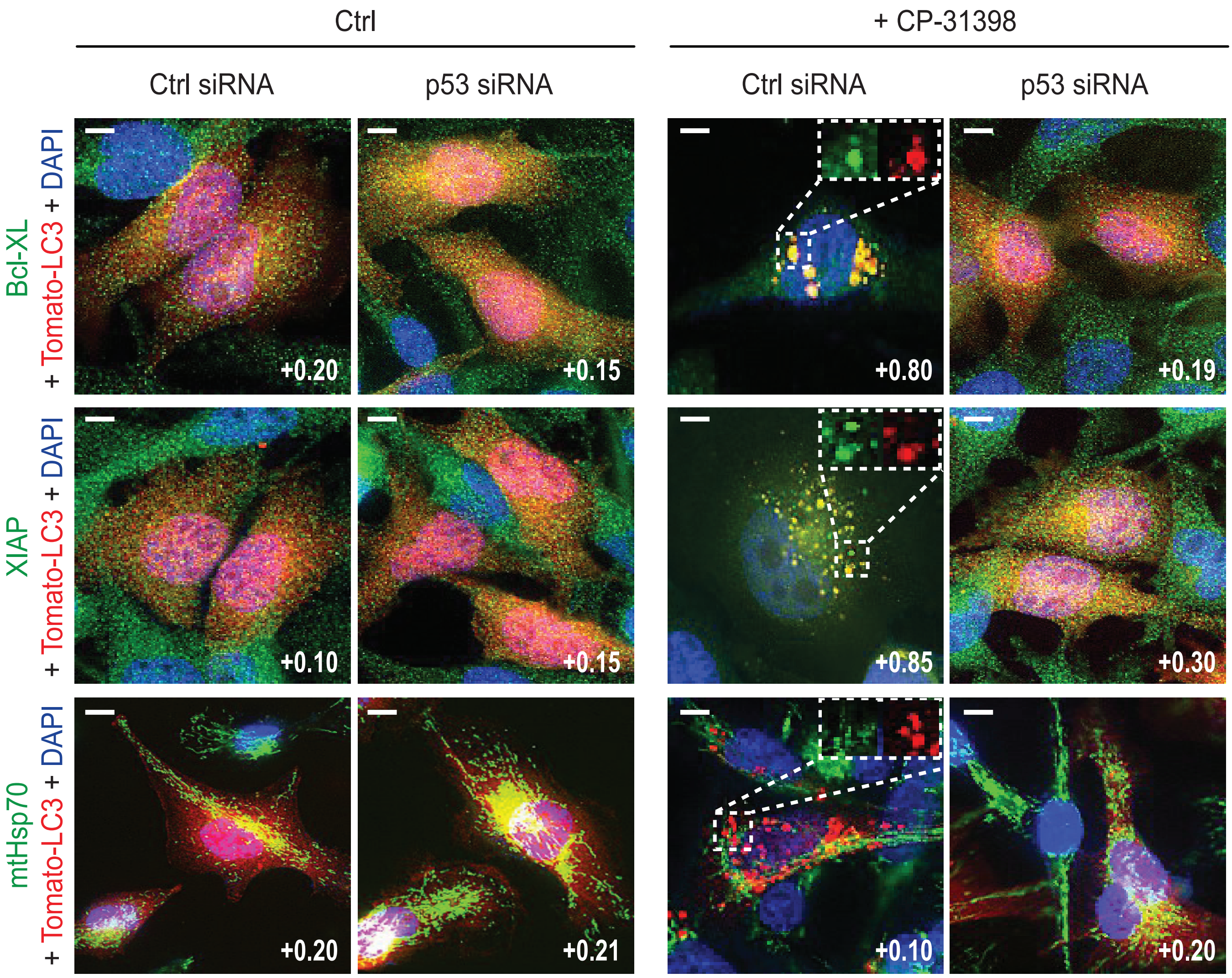

B

Number of Tomato-LC3 + autophagosomes per cell (mean+/-SD)

Ctrl siRNA	0.2 +/- 0.2	N.S.
P53 siRNA	1.3 +/- 0.3	
Ctrl siRNA +CP-31398	18.3 +/- 8.9	*
P53 siRNA +CP-31398	3.9 +/- 2.5	

C

Colocalization (+CP-31398)		Pearson's correlation coefficient R(r) (mean +/- s.d.)	
Tomato-LC3/Bcl-XL	Ctrl siRNA	+0.85 +/- 0.05	*
	p53 siRNA	+ 0.28 +/- 0.10	
Tomato-LC3/XIAP	Ctrl siRNA	+ 0.75 +/- 0.15	*
	p53 siRNA	+ 0.23 +/- 0.13	
Tomato-LC3/mtHsp70	Ctrl siRNA	+ 0.25 +/- 0.15	N.S.
	p53 siRNA	+ 0.27 +/- 0.12	

Figure S7. CP-31398 induced sequestration of Bcl-X_L and XIAP in autophagosomes is inhibited in p53 siRNA treated cells

(A) CP-31398-induced Tomato-$LC3^+$ autophagosomes contains the anti-apoptotic proteins Bcl-X_L and XIAP, but not the mitochondrial marker mtHsp70, in control siRNA-treated cells. After mutant p53 knockdown using siRNA, autophagosomes are mostly undetectable in response to CP-31398 and Bcl-X_L and XIAP are no longer associated with $LC3^+$ structures. Scale bars: 20 μm. Data are representative of three independent fluorescence microscopy experiments. **(B)** The inhibition of p53 expression using siRNA strongly decrease the number of LC3-$Tomato^+$ autophagosomes formed after CP-31398 treatment, counted from microscopy images (100 cells for each conditions) **(C)** The colocalization between CP-31398-induced Tomato-$LC3^+$ autophagosomes and the indicated proteins (stained in green) was measured and displayed as a pearson's correlation coefficient R(r) (also indicated as a number in (A)). The data (mean ± s.d.) obtained from 20 images containing at least 5 cells from three independent experiments are shown. Bcl-X_L and XIAP staining (R(r) > 0.6) is strongly associated with Tomato-$LC3^+$ autophagosomes. Data are representative of three independent experiments (A) or are the mean ± s.d. of three independent experiments (B-C). The p values (B-C) were were determined by unpaired two-tailed Student t test. $*P < 0.05$.

Figure S8

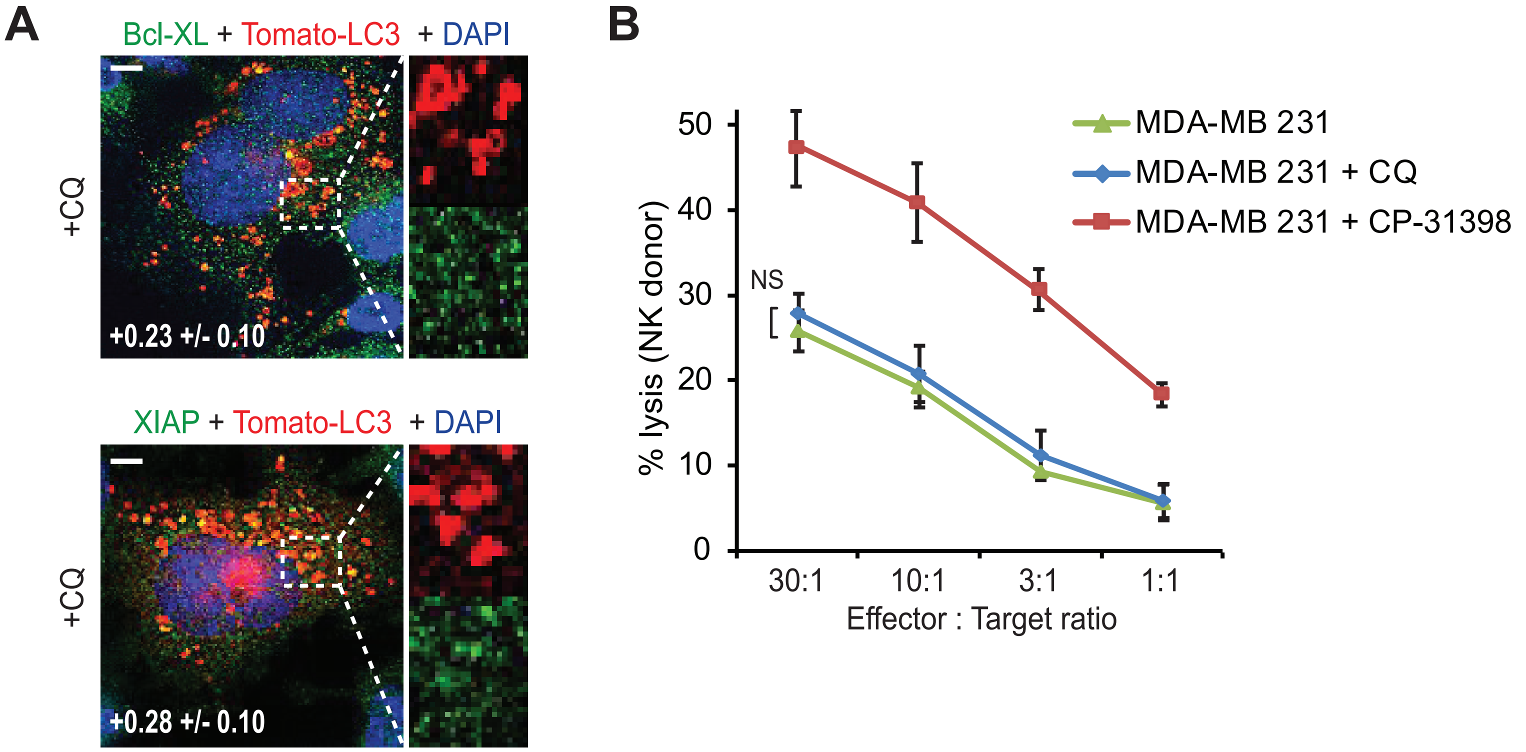
A
Bcl-XL + Tomato-LC3 + DAPI
+CQ
+0.23 +/- 0.10
XIAP + Tomato-LC3 + DAPI
+CQ
+0.28 +/- 0.10
B
MDA-MB 231
MDA-MB 231 + CQ
MDA-MB 231 + CP-31398
% lysis (NK donor)
NS
50
40
30
20
10
0
30:1
10:1
3:1
1:1
Effector : Target ratio

Figure S8. Chloroquine-induced autophagosomes do not sequestrate Bcl-X_L and XIAP and do not increases MDA-MB231 cell susceptibility to NK-mediated lysis

(A) Chloroquine (CQ)-induced Tomato-LC3$^+$ autophagosomes do not contains the anti-apoptotic proteins Bcl-X_L and XIAP. Scale bars: 20 μm. Data are representative of two independent fluorescence microscopy experiments. The colocalization between CQ-induced Tomato-LC3$^+$ autophagosomes and the indicated proteins (stained in green) was measured and displayed as a pearson's correlation coefficient R(r) (mean ± s.d. of two independent experiments). **(B)** CQ pre-treatment (16 hrs) does not increase MDA-MB231 cell susceptibility to NK-mediated lysis. ^{51}Cr release assay using NK cells isolated from a healthy donor co-cultured with target cells at different E:T ratios is shown. Data are the mean ± s.d. of two independent experiments performed in triplicate. The p values (NS: non-significant) were determined by unpaired two-tailed Student t test.

Figure S9

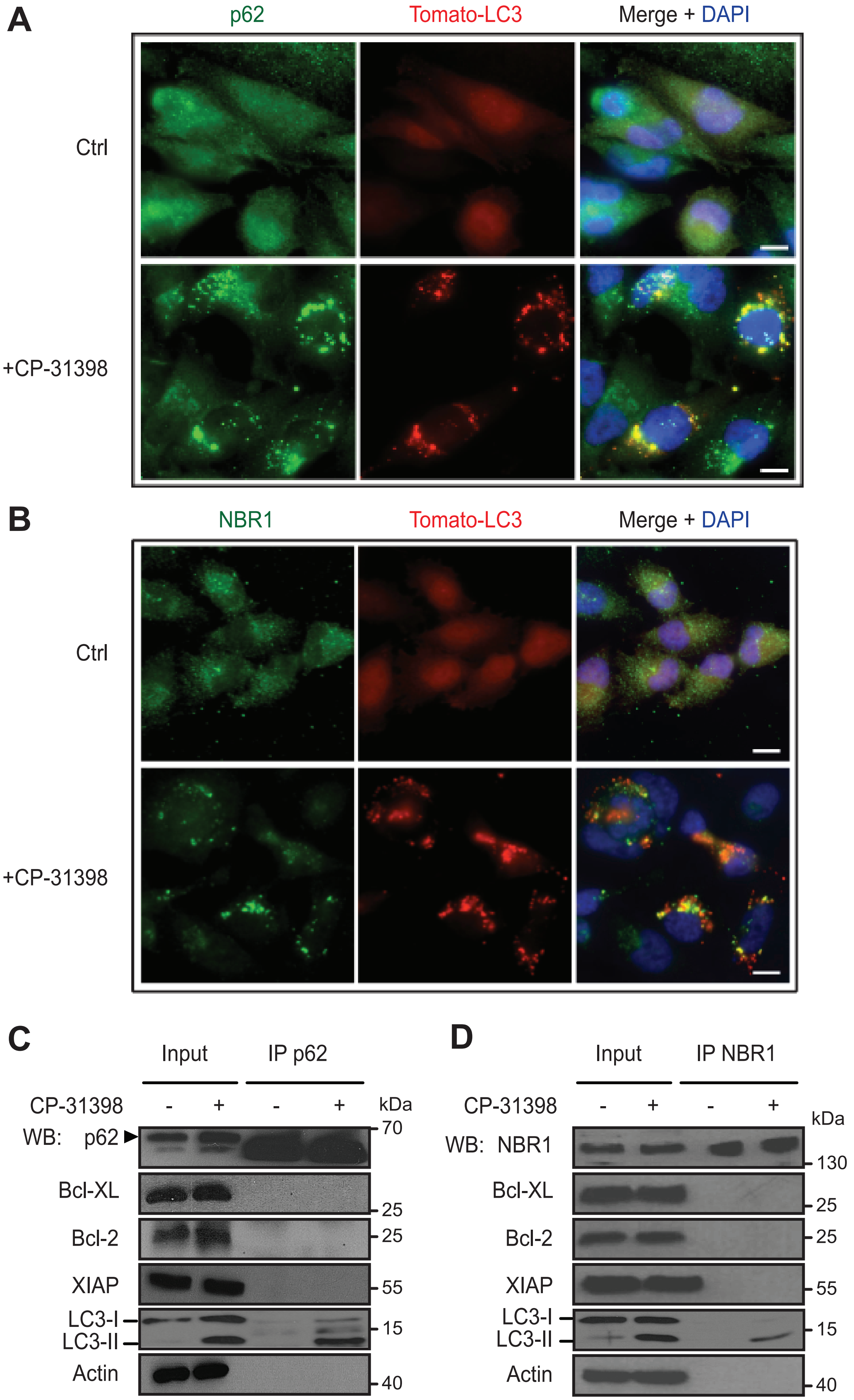
A
p62
Tomato-LC3
Merge + DAPI
Ctrl
+CP-31398
B
NBR1
Tomato-LC3
Merge + DAPI
Ctrl
+CP-31398
C
Input
IP p62
CP-31398
- + - +
kDa
WB: p62
Bcl-XL
Bcl-2
XIAP
LC3-I
LC3-II
Actin
70
25
25
55
15
40
D
Input
IP NBR1
CP-31398
- + - +
kDa
WB: NBR1
Bcl-XL
Bcl-2
XIAP
LC3-I
LC3-II
Actin
130
25
25
55
15
40

Figure S9. The autophagosomal sequestration of Bcl-X_L, Bcl-2 and XIAP in response to CP-31398 is independent of p62 and NBR1

(A-B) P62 and NBR1 relocalize to LC3-Tomato$^+$ autophagosomes formed in response to CP-31398 treatment (48 hrs). Representative fluorescence microscopy images from two independent experiments are displayed. Scale bars: 10 µm. **(C-D)** P62 or NBR1 do not interact with Bcl-X_L, Bcl-2 and XIAP in response to CP-31398. P62 and NBR1 were independently immuno-precipitated from MDA-MB231 cells after 24 hrs treatment with CP-31398 before western-blot analysis of Bcl-X_L, Bcl-2 and XIAP. Immunoblotting for LC3-II was used as a positive control. Data are representative of three independent experiments.
